# Supplementary material for: GENEVIC: GENetic data Exploration and Visualization via Intelligent interactive Console
Source: Bioinformatics. 2024 Aug 8;40(10):btae500. doi: 10.1093/bioinformatics/btae500 (PMC11467054; doi:10.1093/bioinformatics/btae500)
Supplement: btae500_Supplementary_Data [file btae500_supplementary_data.zip › Supplementary_VideoNavigateLink.pdf]

To navigate GENEVIC, refer to our video tutorial (https://tinyurl.com/VideoNavigate)
