# Supplementary material for: GENEVIC: GENetic data Exploration and Visualization via Intelligent interactive Console
Source: Bioinformatics. 2024 Aug 8;40(10):btae500. doi: 10.1093/bioinformatics/btae500 (PMC11467054; doi:10.1093/bioinformatics/btae500)
Supplement: btae500_Supplementary_Data [file btae500_supplementary_data.zip › Supplementary_Documenation_TestCasesExlained.pdf]

GENEVIC

GENetic data Exploration and Visualization via Intelligent interactive Console

Author & Presenter: Anindita Nath

SBMI, UTHealth Houston

Dated: Monday, June 24, 2024

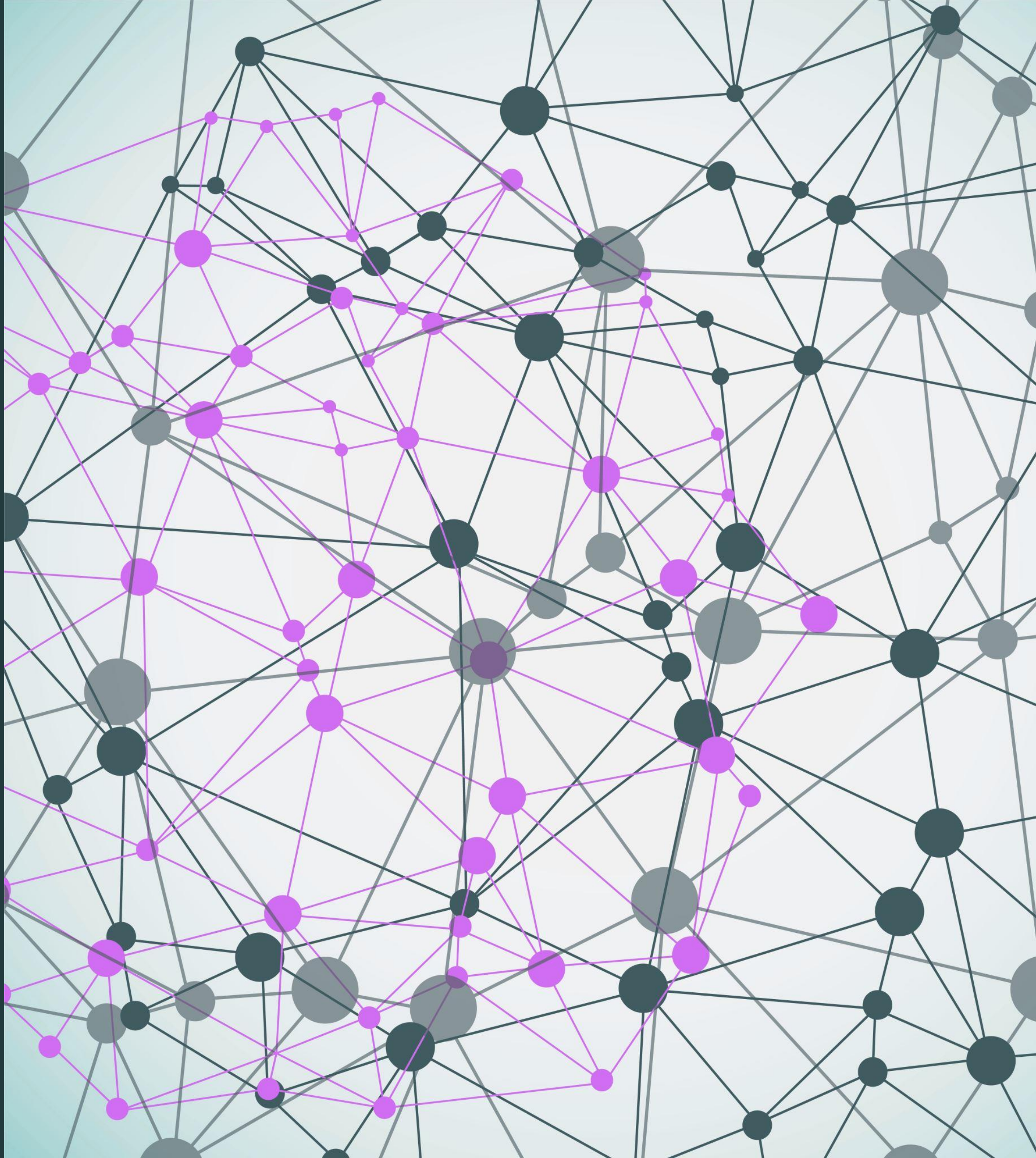

Motivation

- ✓ Surge in biomedical knowledge and tools
- ✓ Knowledge discovery a challenge, requires deep domain and data science expertise
- ✓ Generative AI (Chat GPT, GPT-4) ‘hallucination effect’, unreliable in specialized fields.

GENEVIC: key features

- ✓ enable dynamic engagement with the PolyGenic Score(PGS) Catalog
  - enhancing data mining and discerning intricate patterns
  - broadens access to domain expertise

- ✓ integrates bioinformatics APIs, STRING and ENRICHR:
  - enriching polygenic score analysis with specialized knowledge
  - identification of genetic markers and pathways.
- ✓ supports literature searches from major sites

- ✓ facilitates detailed analysis and informed decision-making
- ✓ democratizes access to genetic research insights
- ✓ simplifying data for diverse research backgrounds
- ✓ promoting multidisciplinary collaboration

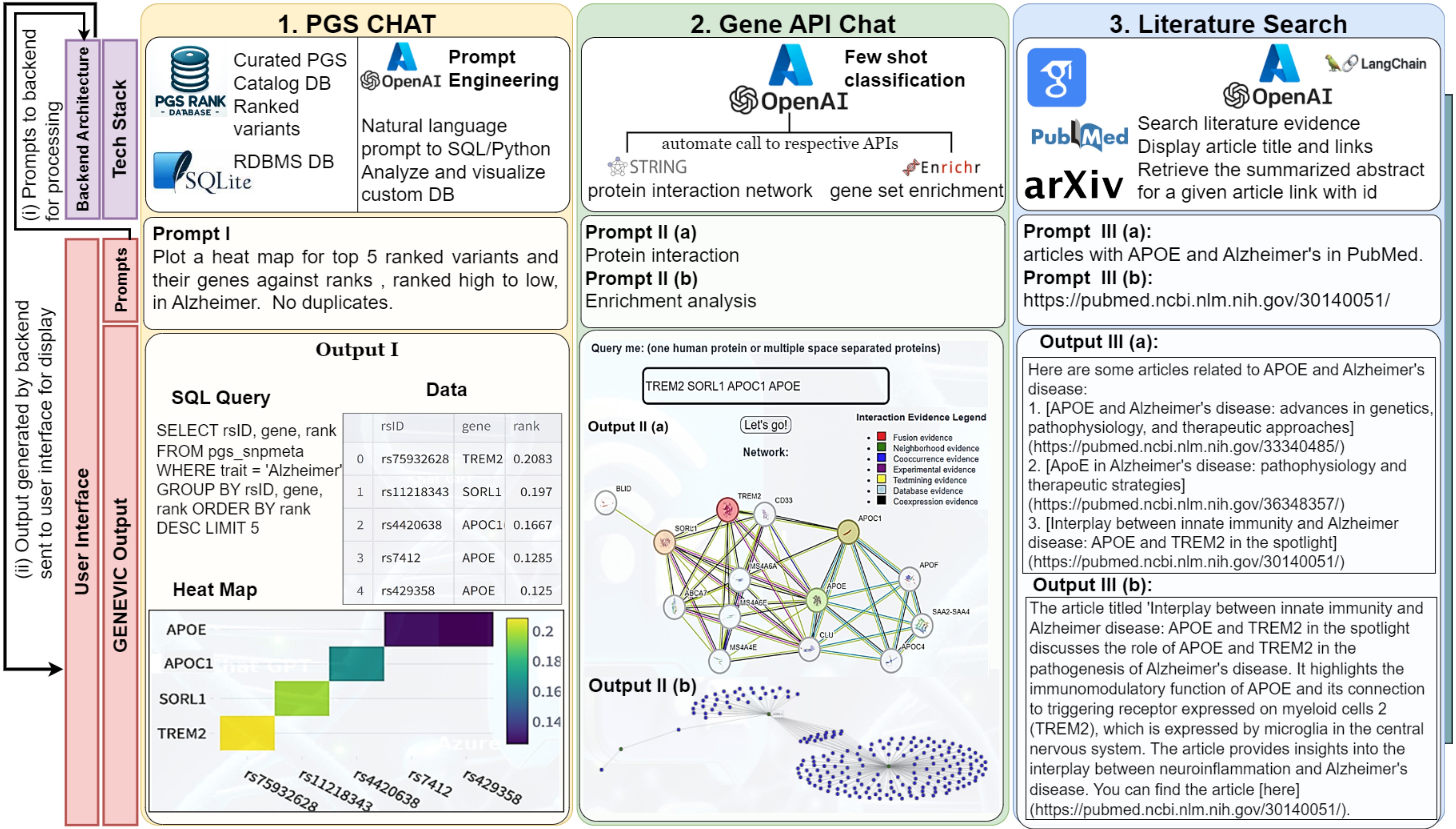
1. PGS CHAT
Curated PGS Catalog DB Ranked variants
PGS RANK - DATABASE -
RDBMS DB
SQLite
OpenAI
Prompt Engineering
Natural language prompt to SQL/Python
Analyze and visualize custom DB
Prompt I
Plot a heat map for top 5 ranked variants and their genes against ranks , ranked high to low, in Alzheimer. No duplicates.
Output I
SQL Query
SELECT rsID, gene, rank FROM pgs_snpmeta WHERE trait = 'Alzheimer' GROUP BY rsID, gene, rank ORDER BY rank DESC LIMIT 5
Data
rsID gene rank
0 rs75932628 TREM2 0.2083
1 rs11218343 SORL1 0.197
2 rs4420638 APOC1 0.1667
3 rs7412 APOE 0.1285
4 rs429358 APOE 0.125
Heat Map
APOE
APOC1
SORL1
TREM2
rs75932628
rs11218343
rs4420638
rs7412
rs429358
0.2
0.18
0.16
0.14
2. Gene API Chat
OpenAI
Few shot classification
automate call to respective APIs
STRING
Enrichr
protein interaction network
gene set enrichment
Prompt II (a)
Protein interaction
Prompt II (b)
Enrichment analysis
Query me: (one human protein or multiple space separated proteins)
TREM2 SORL1 APOC1 APOE
Output II (a)
Let's go!
Network:
Interaction Evidence Legend
Fusion evidence
Neighborhood evidence
Cooccurrence evidence
Experimental evidence
Textmining evidence
Database evidence
Coexpression evidence
BLID
TREM2
CD33
SORL1
APOC1
MS4A6A
APOF
ABCA7
APOE
MS4A6E
SAA2-SAA4
CLU
MS4A4E
APOC4
Output II (b)
3. Literature Search
LangChain
OpenAI
PubMed
arXiv
Search literature evidence
Display article title and links
Retrieve the summarized abstract for a given article link with id
Prompt III (a):
articles with APOE and Alzheimer's in PubMed.
Prompt III (b):
https://pubmed.ncbi.nlm.nih.gov/30140051/
Output III (a):
Here are some articles related to APOE and Alzheimer's disease:
1. [APOE and Alzheimer's disease: advances in genetics, pathophysiology, and therapeutic approaches] (https://pubmed.ncbi.nlm.nih.gov/33340485/)
2. [ApoE in Alzheimer's disease: pathophysiology and therapeutic strategies] (https://pubmed.ncbi.nlm.nih.gov/36348357/)
3. [Interplay between innate immunity and Alzheimer disease: APOE and TREM2 in the spotlight] (https://pubmed.ncbi.nlm.nih.gov/30140051/)
Output III (b):
The article titled 'Interplay between innate immunity and Alzheimer disease: APOE and TREM2 in the spotlight discusses the role of APOE and TREM2 in the pathogenesis of Alzheimer's disease. It highlights the immunomodulatory function of APOE and its connection to triggering receptor expressed on myeloid cells 2 (TREM2), which is expressed by microglia in the central nervous system. The article provides insights into the interplay between neuroinflammation and Alzheimer's disease. You can find the article [here] (https://pubmed.ncbi.nlm.nih.gov/30140051/).
(i) Prompts to backend for processing
(ii) Output generated by backend sent to user interface for display
Backend Architecture
Tech Stack
User Interface
Prompts
GENEVIC Output

Custom Database: PolyGenicScore(PGS) Rank Database

- ✓ PGS score files from PGS Catalog (Release: August 4th, 2023), human reference genome (GRCh38 build), local database for easier access.
- ✓ Trait specific data using R's "Quincunx" package, querying the catalog's REST API with ontology IDs
- ✓ essential columns: effect allele, effect weight, and SNP rsID/SNP coordinates, and harmonized variant labeling discrepancies.
- ✓ merged phenotype specific PGS files into a single dataset using R's "dplyr" and "bind_rows", missing values as 'NA'
- ✓ ranked variants using the *Dowdall* method, an alternate Borda method, based on their absolute effect weights and annotated the variants using ANNOVAR:
    - assigning the reciprocal of ranks (RR) to each variant
    - top rank -> RR is 1, 2nd rank -> RR is 1/2, 3rd rank -> RR is 1/3, so on.
    - For each variant query (Q), calculate the mean of the reciprocals of its ranks (MRR) across multiple scores. weights unavailable -> MRR of 0.

PolyGenicScore(PGS) Rank Database (contd.)

Phenotype	#PGSFiles	SNPINfo
Alzheimer	23	w:[(-0.95)-1.64]; m:0.0013; md: 5.82e-07; sd: 0.0390
Schizophrenia	5	w:[(-0.04)-0.05]; m:5.61e-05; md:8.36e-06; sd:0.0018
Cognition	5	w:[(-3.60)-1.68]; m:(-0.0005); md:-7e-08;sd:0.0321

Table 1: Summary of statistics of the phenotypes used to develop the PGS Rank database. Phenotype denotes disease or trait name, #PGSFiles denote the count of score files for each PGS Score ID corresponding to the phenotype, SNP info refers to the aggregated information regarding the SNPs or variants in each score file. w is the weight range, m, md, sd denote the mean, median, and standard deviation of weights, respectively.

Availability

- ✓ Streamlit community cloud at *https://genevic-anath2024.streamlit.app*, ready to use without installation.
  - Streamlit's 1 GB data limit, PGS Rank DB is pared down to top 100 genes each for Alzheimer's, schizophrenia, and cognition
  - adequate for this pilot project but should be expanded later with architecture of higher capacity
- ✓ runnable locally, as detailed in the installation guide, *http://tinyurl.com/InstalaltionGuide*
  - prerequisite being Python 3.10 or higher version

Home Page

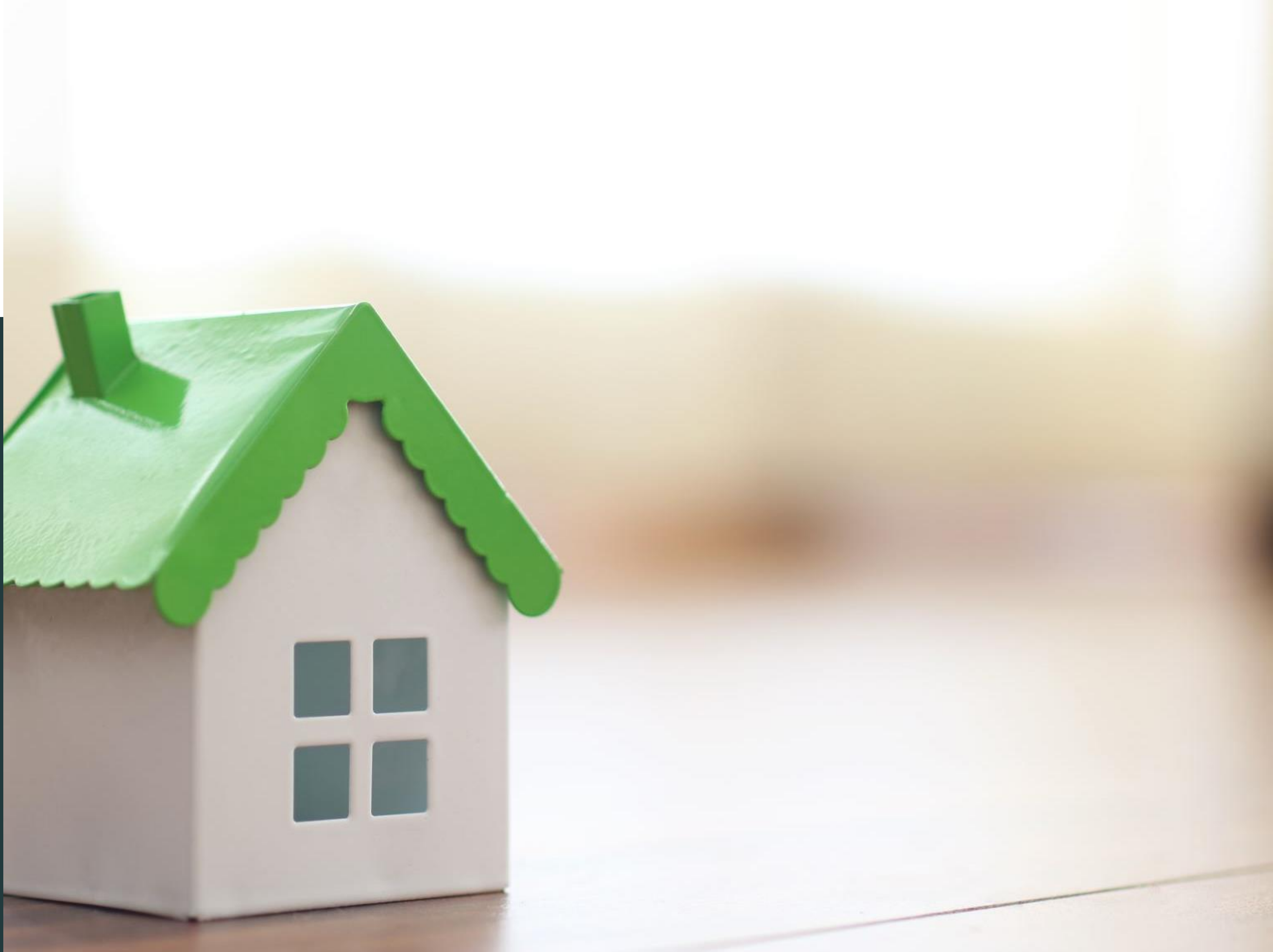

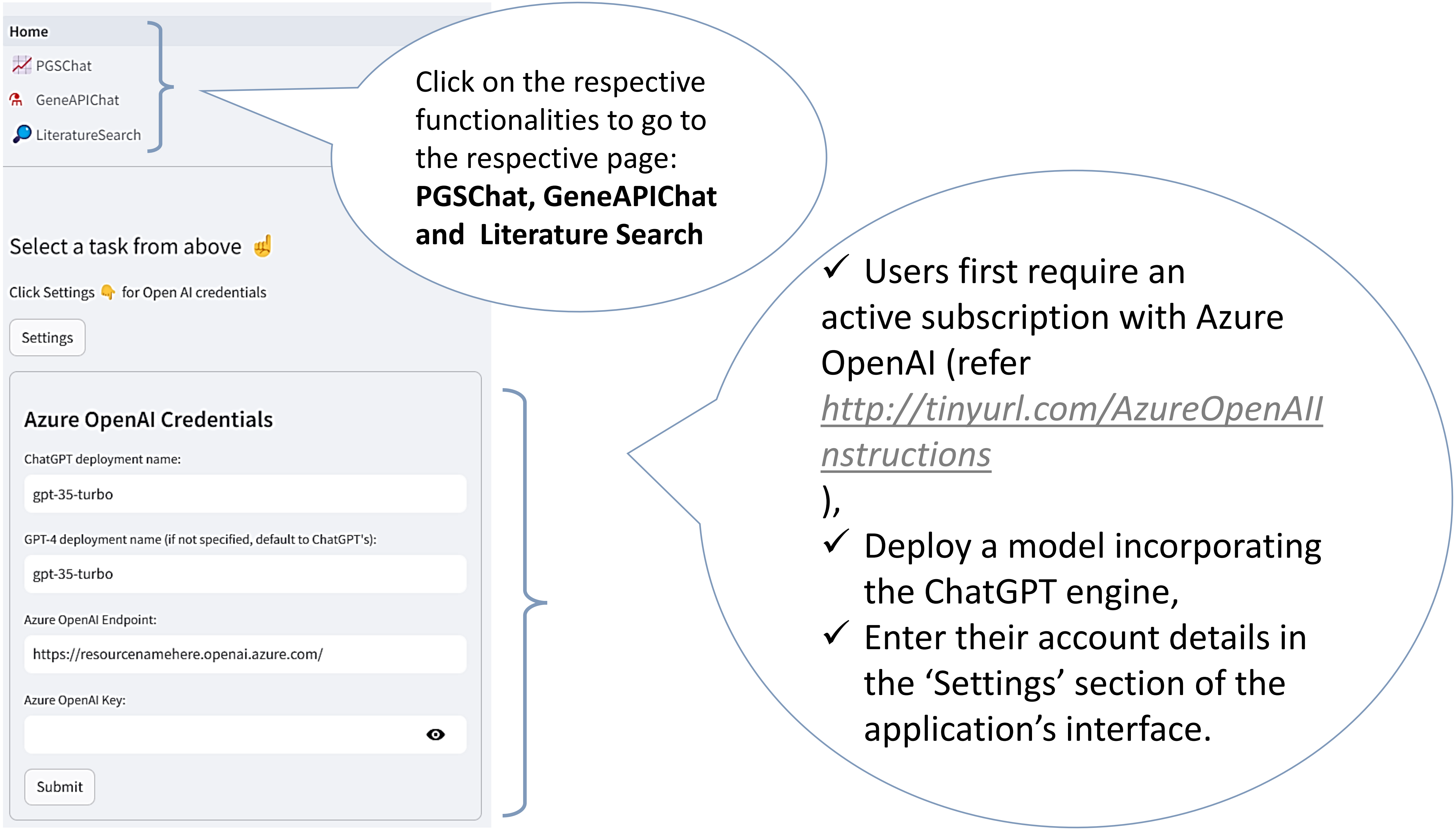
Home
PGSChat
GeneAPIChat
LiteratureSearch
Select a task from above
Click Settings for Open AI credentials
Settings
Azure OpenAI Credentials
ChatGPT deployment name:
gpt-35-turbo
GPT-4 deployment name (if not specified, default to ChatGPT's):
gpt-35-turbo
Azure OpenAI Endpoint:
https://resourcenamehere.openai.azure.com/
Azure OpenAI Key:
Submit
Click on the respective functionalities to go to the respective page: PGSChat, GeneAPIChat and Literature Search
✓ Users first require an active subscription with Azure OpenAI (refer http://tinyurl.com/AzureOpenAIInstructions),
✓ Deploy a model incorporating the ChatGPT engine,
✓ Enter their account details in the 'Settings' section of the application's interface.

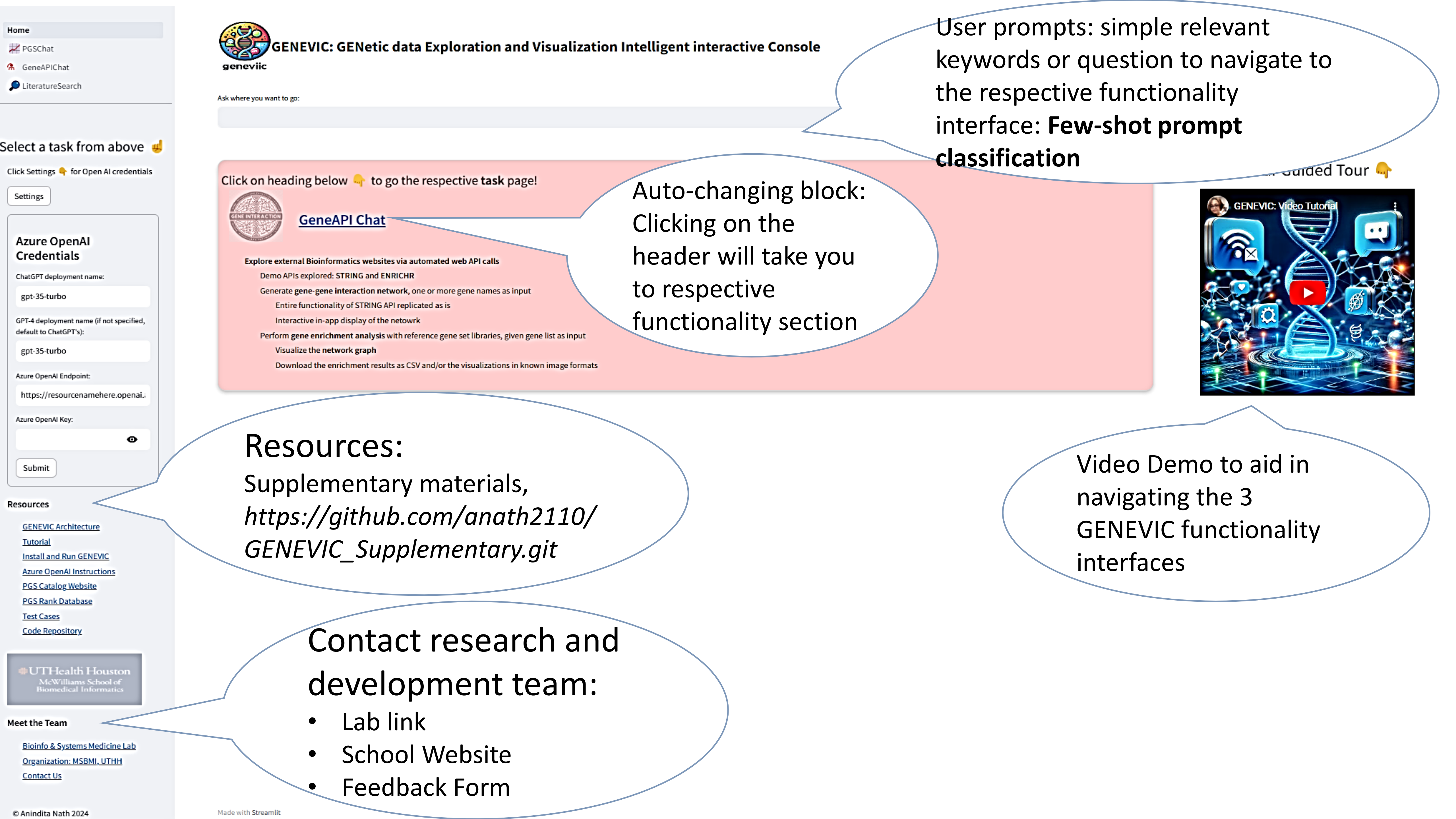
Home
PGSChat
GeneAPIChat
LiteratureSearch
Select a task from above
Click Settings for Open AI credentials
Settings
Azure OpenAI Credentials
ChatGPT deployment name:
gpt-35-turbo
GPT-4 deployment name (if not specified, default to ChatGPT's):
gpt-35-turbo
Azure OpenAI Endpoint:
https://resourcenamehere.openai.
Azure OpenAI Key:
Submit
Resources
GENEVIC Architecture
Tutorial
Install and Run GENEVIC
Azure OpenAI Instructions
PGS Catalog Website
PGS Rank Database
Test Cases
Code Repository
UTHealth Houston McWilliams School of Biomedical Informatics
Meet the Team
Bioinfo & Systems Medicine Lab
Organization: MSBMI, UTHH
Contact Us
© Anindita Nath 2024
geneviic
GENEVIC: GENetic data Exploration and Visualization Intelligent interactive Console
Ask where you want to go:
Click on heading below to go the respective task page!
GENE INTERACTION
GeneAPI Chat
Explore external Bioinformatics websites via automated web API calls
Demo APIs explored: STRING and ENRICHR
Generate gene-gene interaction network, one or more gene names as input
Entire functionality of STRING API replicated as is
Interactive in-app display of the netowrk
Perform gene enrichment analysis with reference gene set libraries, given gene list as input
Visualize the network graph
Download the enrichment results as CSV and/or the visualizations in known image formats
Made with Streamlit
Guided Tour
GENEVIC: Video Tutorial
User prompts: simple relevant keywords or question to navigate to the respective functionality interface: **Few-shot prompt classification**
Auto-changing block: Clicking on the header will take you to respective functionality section
Resources:
Supplementary materials, *https://github.com/anath2110/GENEVIC_Supplementary.git*
Video Demo to aid in navigating the 3 GENEVIC functionality interfaces
Contact research and development team:
• Lab link
• School Website
• Feedback Form

PGS Chat

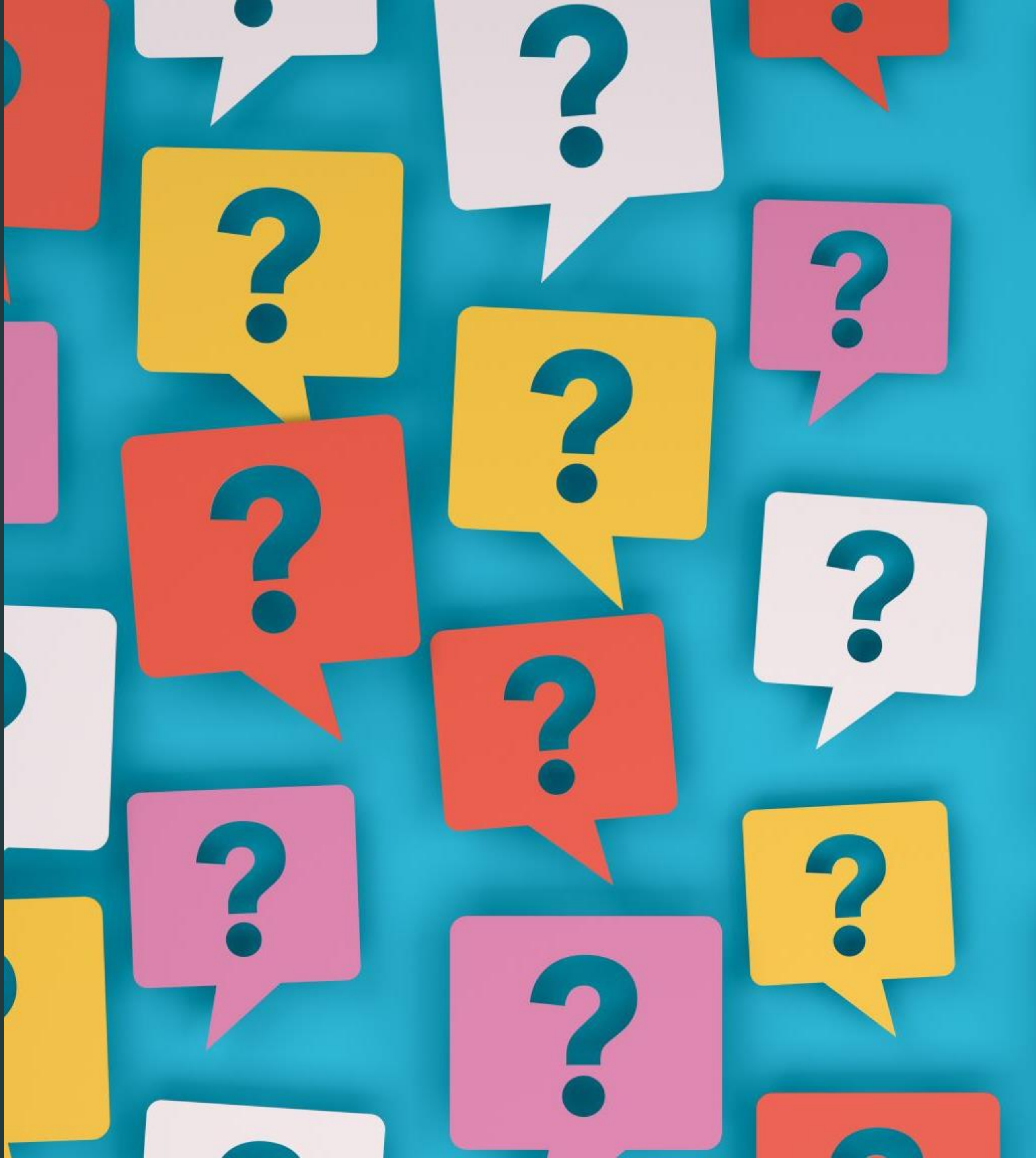

PGS Chat
Test Case 1:
Information Retrieval

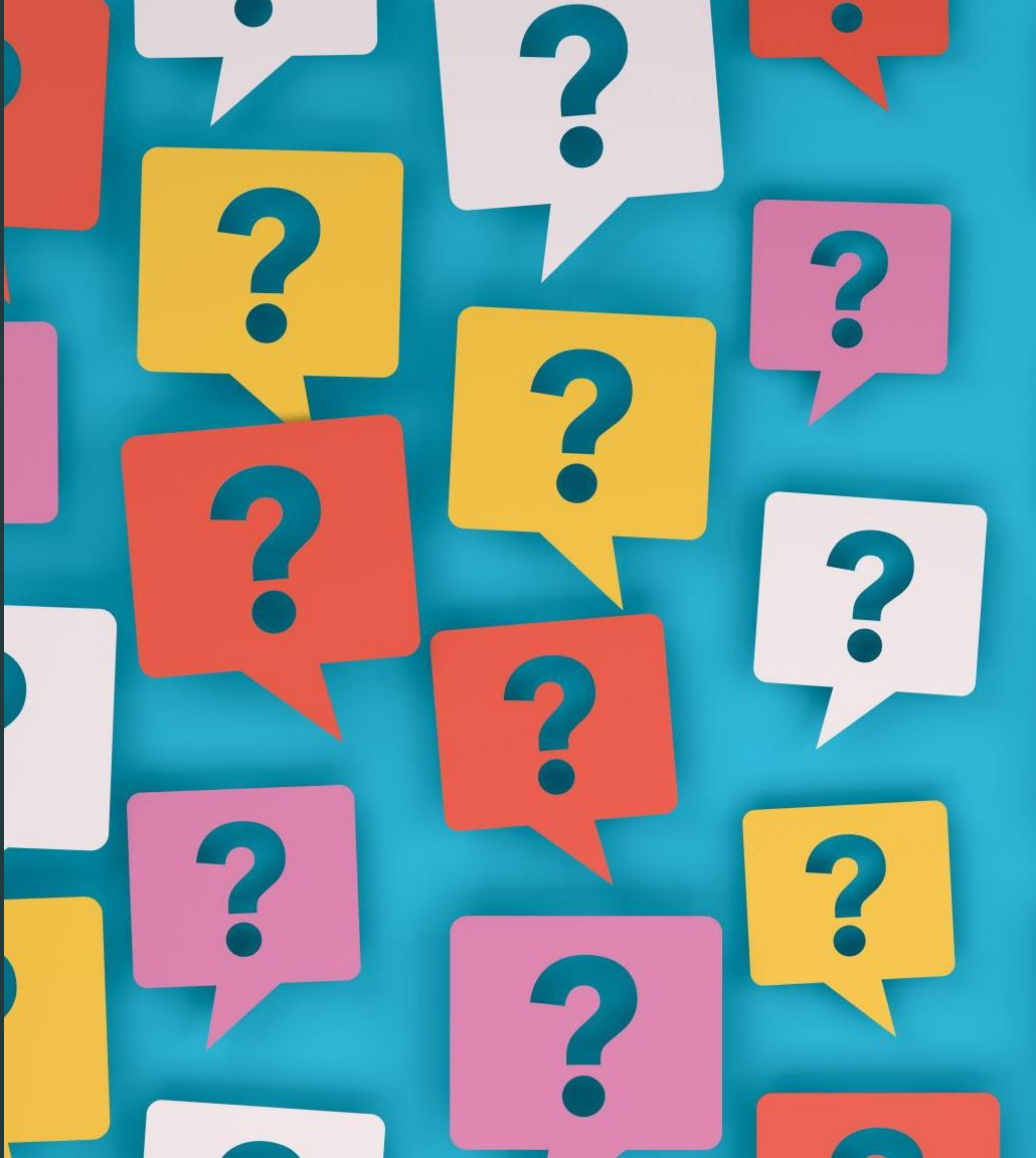

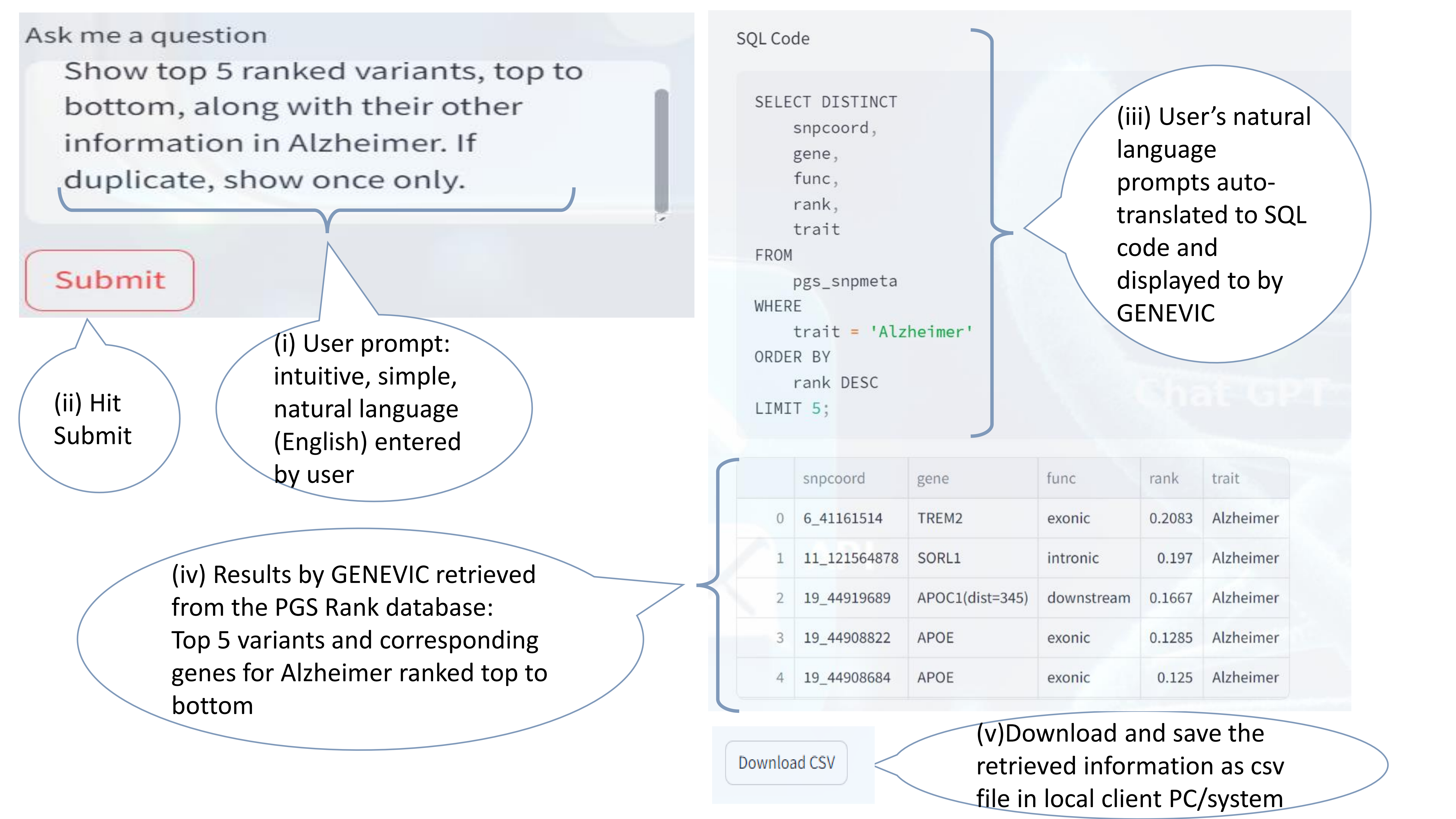
Ask me a question
Show top 5 ranked variants, top to bottom, along with their other information in Alzheimer. If duplicate, show once only.
Submit
(ii) Hit Submit
(i) User prompt: intuitive, simple, natural language (English) entered by user
SQL Code
SELECT DISTINCT
snpcoord,
gene,
func,
rank,
trait
FROM
pgs_snpmeta
WHERE
trait = 'Alzheimer'
ORDER BY
rank DESC
LIMIT 5;
(iii) User's natural language prompts auto-translated to SQL code and displayed to by GENEVIC
snpcoord gene func rank trait
0 6_41161514 TREM2 exonic 0.2083 Alzheimer
1 11_121564878 SORL1 intronic 0.197 Alzheimer
2 19_44919689 APOC1(dist=345) downstream 0.1667 Alzheimer
3 19_44908822 APOE exonic 0.1285 Alzheimer
4 19_44908684 APOE exonic 0.125 Alzheimer
(iv) Results by GENEVIC retrieved from the PGS Rank database: Top 5 variants and corresponding genes for Alzheimer ranked top to bottom
Download CSV
(v)Download and save the retrieved information as csv file in local client PC/system

PGS Chat
Test Case 2:
Information Retrieval and Visualization

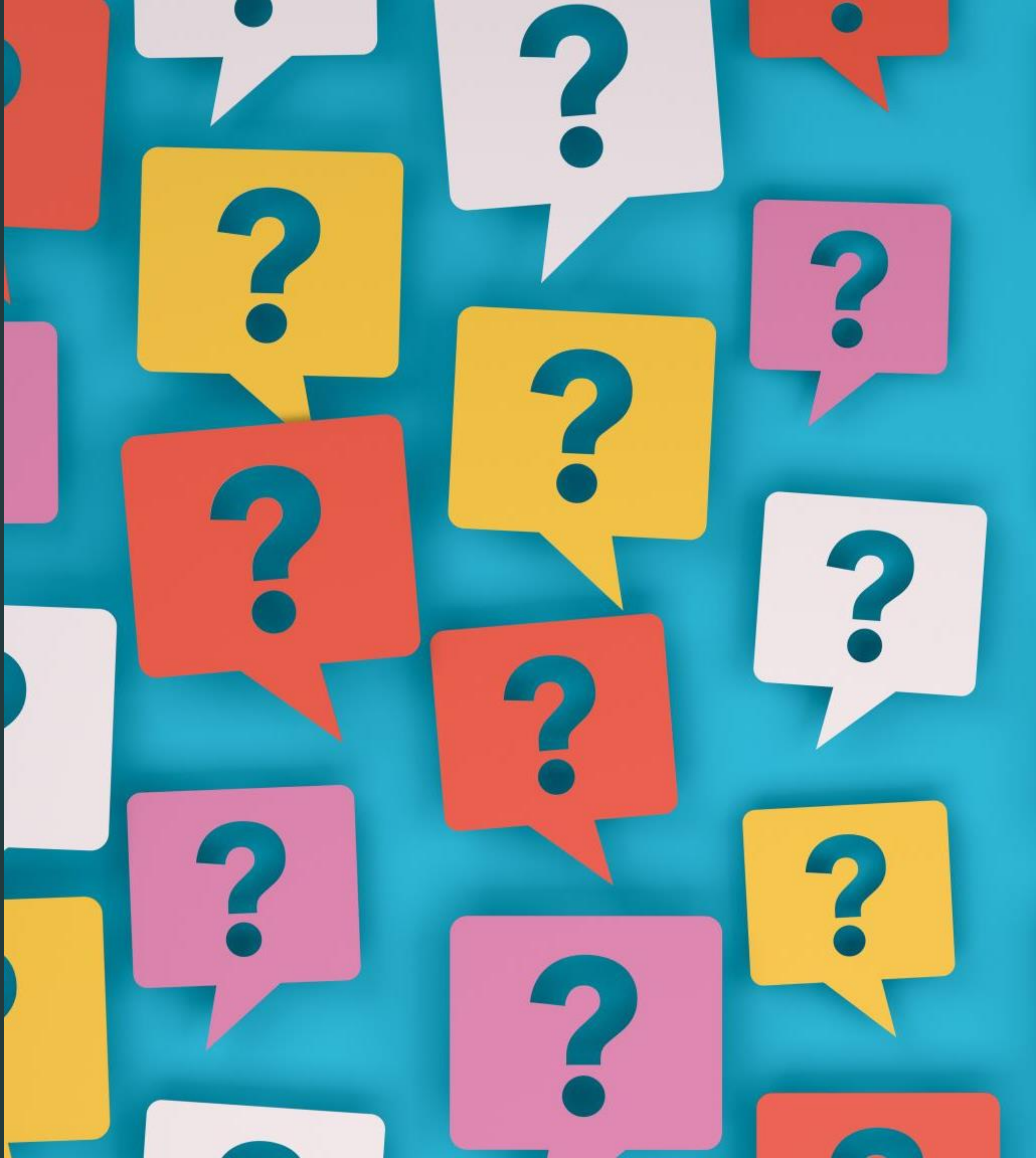

Ask me a question

Plot a heat map for top 5 ranked variants and their genes against ranks , ranked high to low, in Alzheimer. No duplicates.

Submit

(ii) Hit Submit

(i) User prompt: intuitive, simple, natural language (English) entered by user

(iii) User's natural language prompts auto-translated to generate relevant SQL code and displayed to by GENEVIC

Visualize custom database 📈

Question: Plot a heat map for top 5 ranked variants and their genes against ranks , ranked high to low, in Alzheimer. No duplicates.

Thought 1

To plot a heat map for the top 5 ranked variants and their genes against ranks in Alzheimer, we need to retrieve the necessary data from the database. We will need information from the pgs_snpmeta table, specifically the rsID, gene, and rank columns. We will filter the data for Alzheimer and sort it by rank in descending order. We will also remove any duplicate variants.

```
# Query the necessary data
sql_query = "SELECT rsID, gene, rank FROM pgs_snpmeta WHERE trait = 'Alzheimer' GROUP BY rsID, gene, rank
ORDER BY rank DESC LIMIT 5"
data_df = execute_sql(sql_query)

# Replace NAN with 0
data_df = data_df.replace(np.nan, 0)

# Observe the query result
observe("Data", data_df)
```

(iv) Results by GENEVIC retrieved from the PGS Rank database

Data

	rsID	gene	rank
0	rs75932628	TREM2	0.2083
1	rs11218343	SORL1	0.197
2	rs4420638	APOC1(	0.1667
3	rs7412	APOE	0.1285
4	rs429358	APOE	0.125

..Contd..

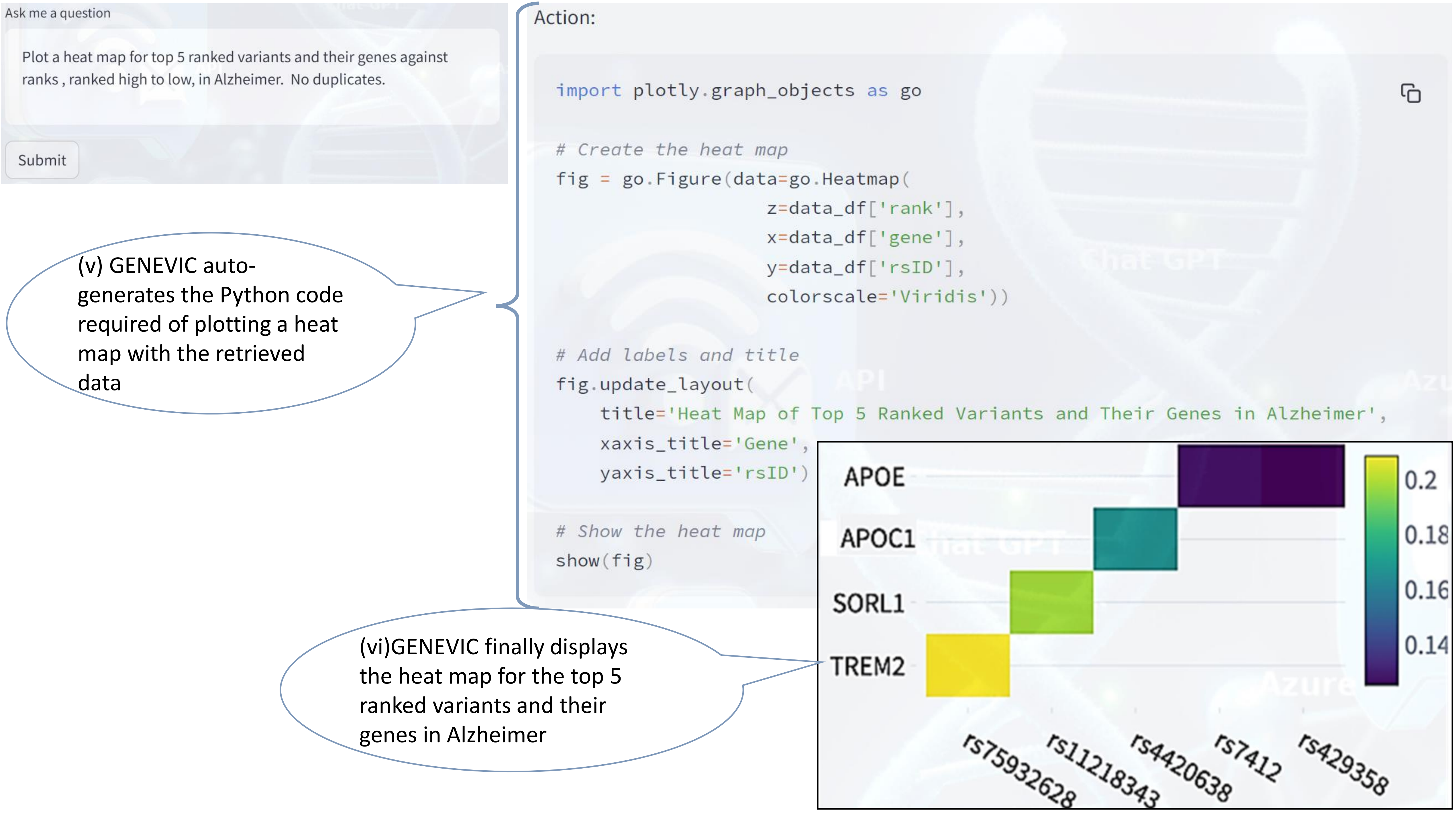
Ask me a question
Plot a heat map for top 5 ranked variants and their genes against ranks , ranked high to low, in Alzheimer. No duplicates.
Submit
(v) GENEVIC auto-generates the Python code required of plotting a heat map with the retrieved data
Action:
import plotly.graph_objects as go
# Create the heat map
fig = go.Figure(data=go.Heatmap(
z=data_df['rank'],
x=data_df['gene'],
y=data_df['rsID'],
colorscale='Viridis'))
# Add labels and title
fig.update_layout(
title='Heat Map of Top 5 Ranked Variants and Their Genes in Alzheimer',
xaxis_title='Gene',
yaxis_title='rsID')
# Show the heat map
show(fig)
(vi)GENEVIC finally displays the heat map for the top 5 ranked variants and their genes in Alzheimer
APOE
APOC1
SORL1
TREM2
rs75932628
rs11218343
rs4420638
rs7412
rs429358
0.2
0.18
0.16
0.14

PGS Chat
Test Case 3: Information Retrieval and Visualization

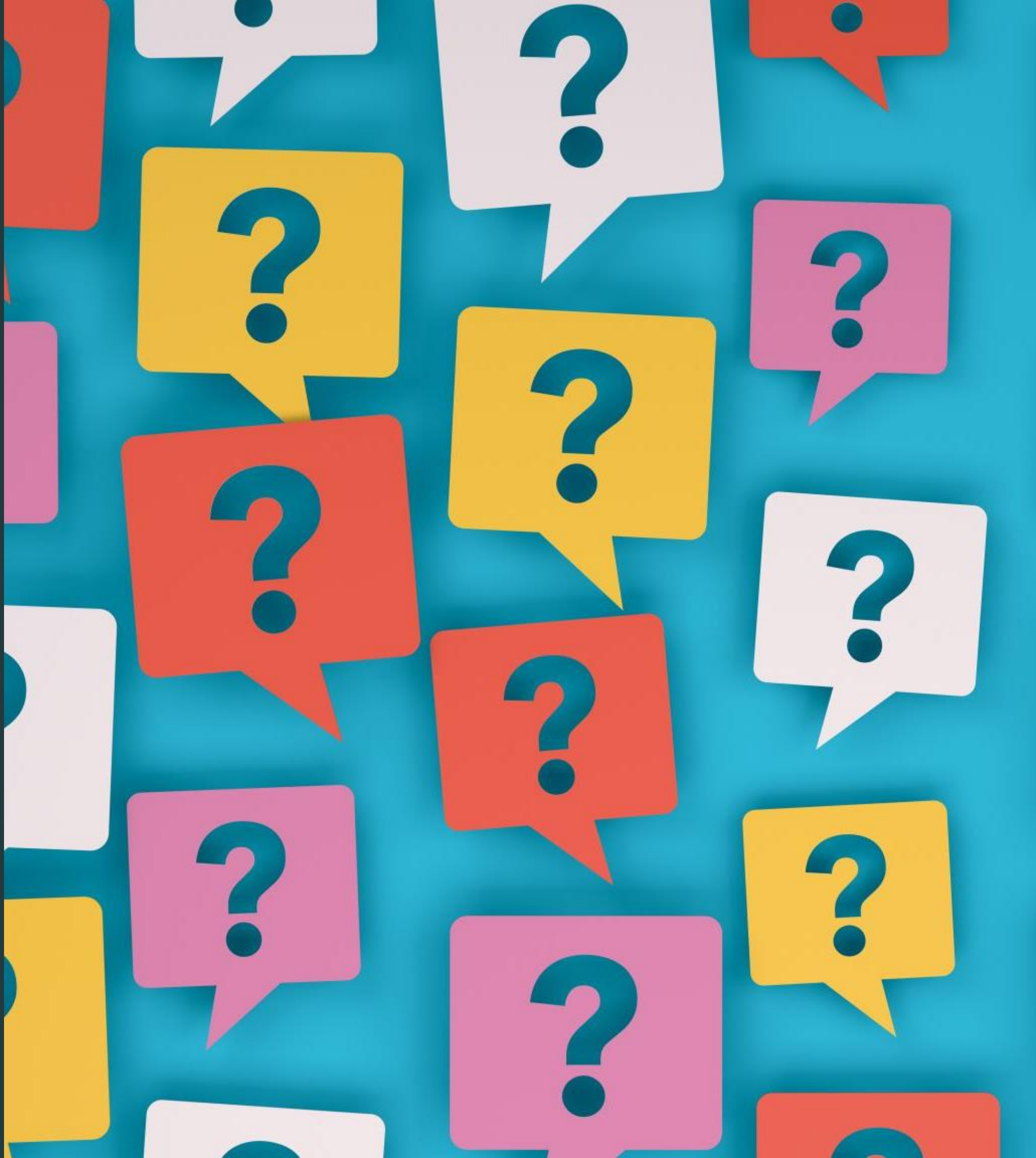

Ask me a question

Plot a heat map with variants against ranks for the top 5 ranked genes, ranked high to low, in Alzheimer. use pgssnpmeta only. If duplicate show only once.

Submit

(ii) Hit Submit

(i) User prompt: intuitive, simple, natural language (English) entered by user

(iii) User's natural language prompts auto-translated to generate relevant SQL code and displayed to by GENEVIC

Visualize custom database

Question: Plot a heat map with variants against ranks for the top 5 ranked genes, ranked high to low, in Alzheimer. use pgssnpmeta only. If duplicate show only once.

Thought 1

To plot a heat map with variants against ranks for the top 5 ranked genes in Alzheimer, we need to retrieve the necessary data from the pgs_snpmeta table. We will filter the data based on the reported_trait column to select only the variants associated with Alzheimer. Then, we will group the data by the gene column and calculate the average rank for each gene. Finally, we will select the top 5 genes based on their average rank and retrieve the variants associated with these genes.

Action:

```
# Import necessary libraries
import plotly.express as px
import numpy as np

# Query the necessary data
sql_query = "SELECT DISTINCT gene, rank FROM pgs_snpmeta WHERE trait = 'Alzheimer' ORDER BY rank DESC LIMIT 5"
top_genes_df = execute_sql(sql_query)

# Replace NAN with 0
top_genes_df['rank'] = top_genes_df['rank'].replace(np.nan, 0)

# Observe the query result
observe("Top Genes Data", top_genes_df)
```

(iv) Results by GENEVIC retrieved from the PGS Rank database: Top 5 genes for Alzheimer's

Top Genes Data

	gene	rank
0	TREM2	0.2083
1	SORL1	0.197
2	APOC1(dist=345)	0.1667
3	APOE	0.1285
4	APOE	0.125

..Contd..

Ask me a question

Plot a heat map with variants against ranks for the top 5 ranked genes, ranked high to low, in Alzheimer. use pgssnpmeta only. If duplicate show only once.

Submit

Thought 2

Now that we have the top 5 ranked genes associated with Alzheimer, we need to retrieve the variants associated with these genes from the pgs_snpmeta table. We will filter the data based on the selected genes and calculate the average rank for each variant. Then, we will create a heat map with variants against ranks.

Action:

```
# Filter the data based on the selected genes
selected_genes = top_genes_df['gene'].tolist()
sql_query = f"SELECT rsID, gene, rank FROM pgs_snpmeta WHERE trait = 'Alzheimer' AND gene IN {tuple(selected_genes)}"
variants_df = execute_sql(sql_query)

# Calculate the average rank for each variant
variants_df = variants_df.groupby(['rsID', 'gene']).mean().reset_index()
```

(v) Then, GENEVIC generates another SQL code to retrieve the variant information corresponding to the top 5 ranked genes from Alzheimer's

Heat Map Data

rsID	APOC1(dist=345)	APOE	SORL1	TREM2
rs11218343	None	None	0.197	None
rs143332484	None	None	None	0.0729
rs429358	None	0.125	None	None
rs4420638	0.1667	None	None	None
rs7412	None	0.1285	None	None
rs75932628	None	None	None	0.2083
rs769449	None	0.0625	None	None

(vi) Data auto-arranged for plotting in Heat Map by GENEVIC

..Contd..

Ask me a question

Plot a heat map with variants against ranks for the top 5 ranked genes, ranked high to low, in Alzheimer. use pgssnpmeta only. If duplicate show only once.

Submit

```
# Pivot the data to create a heat map
heat_map_df = variants_df.pivot(index='rsID', columns='gene', values='rank')

# Observe the heat map data
observe("Heat Map Data", heat_map_df)

# Create the heat map
fig = px.imshow(heat_map_df, x=heat_map_df.columns, y=heat_map_df.index)
fig.update_layout(title="Heat Map of Variants against Ranks for Top 5 Ranked Genes in Alzheimer")
fig.update_xaxes(title="Genes")
fig.update_yaxes(title="Variants")

# Show the heat map to the user
show(fig)
```

(vii) GENEVIC auto-generates the Python code required of plotting a heat map with the retrieved data

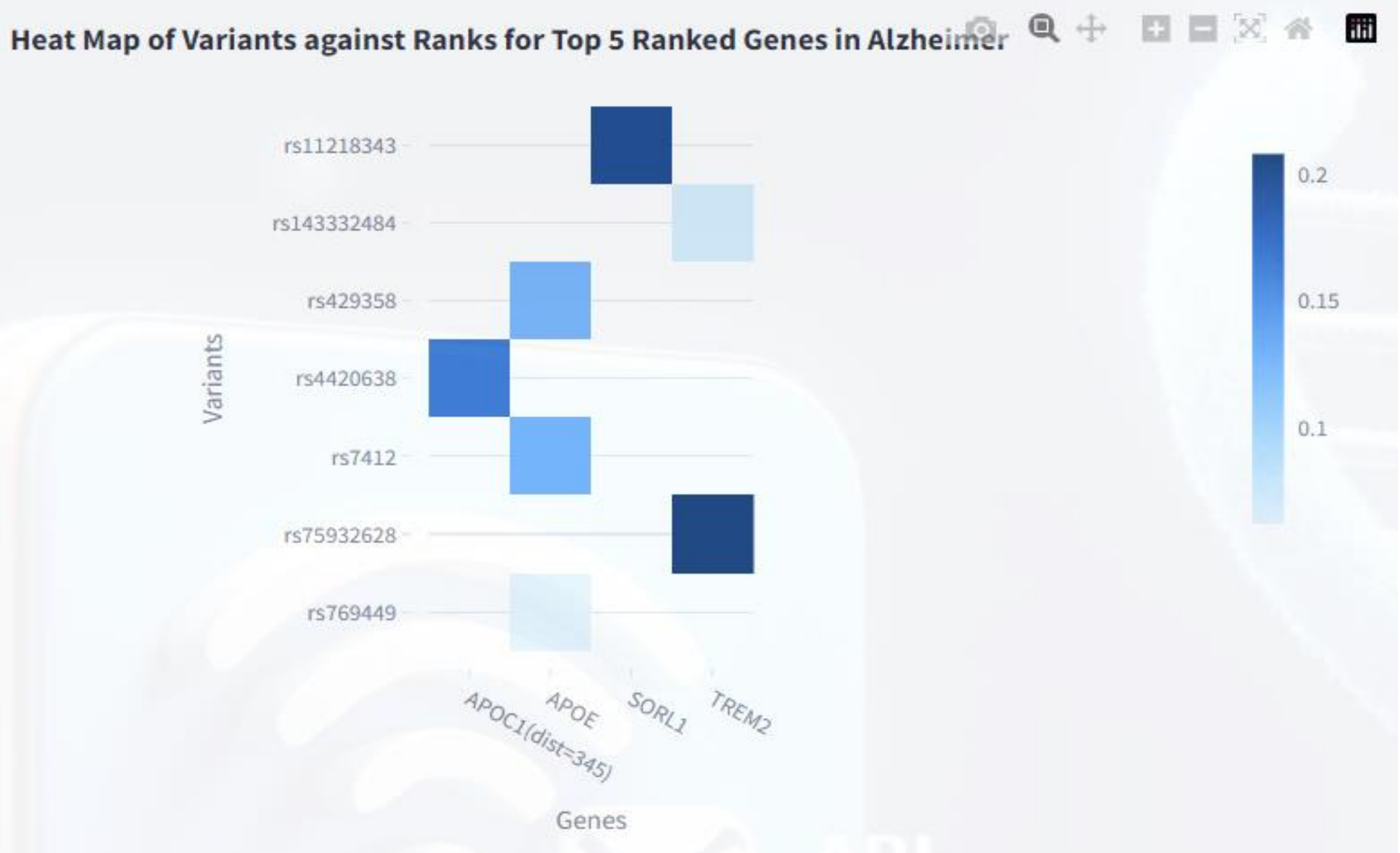

(vii) GENEVIC finally displays the heat map for the top 5 ranked variants and their genes in Alzheimer

PGS Chat
Test Case 4: Information Retrieval and Visualization

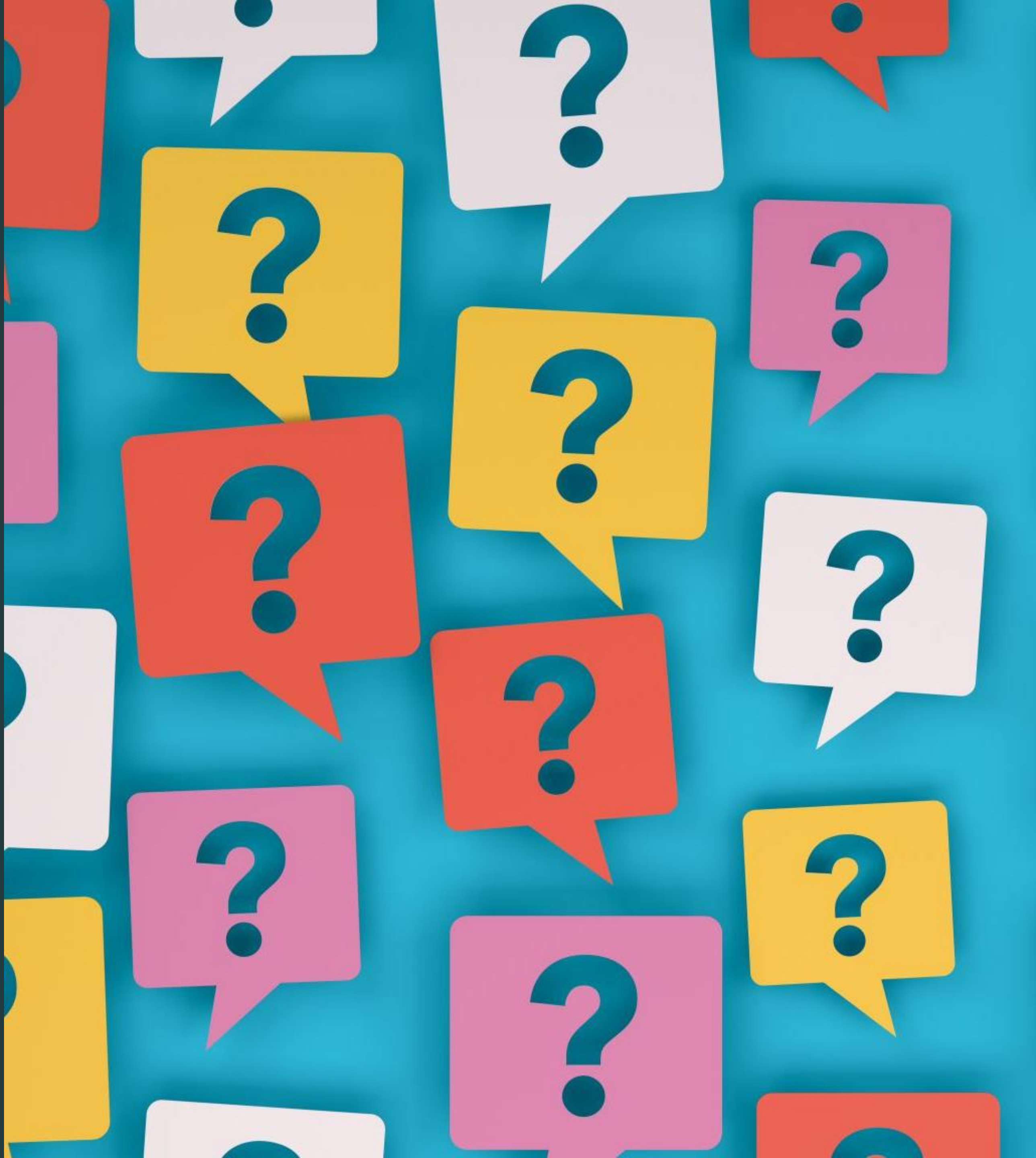

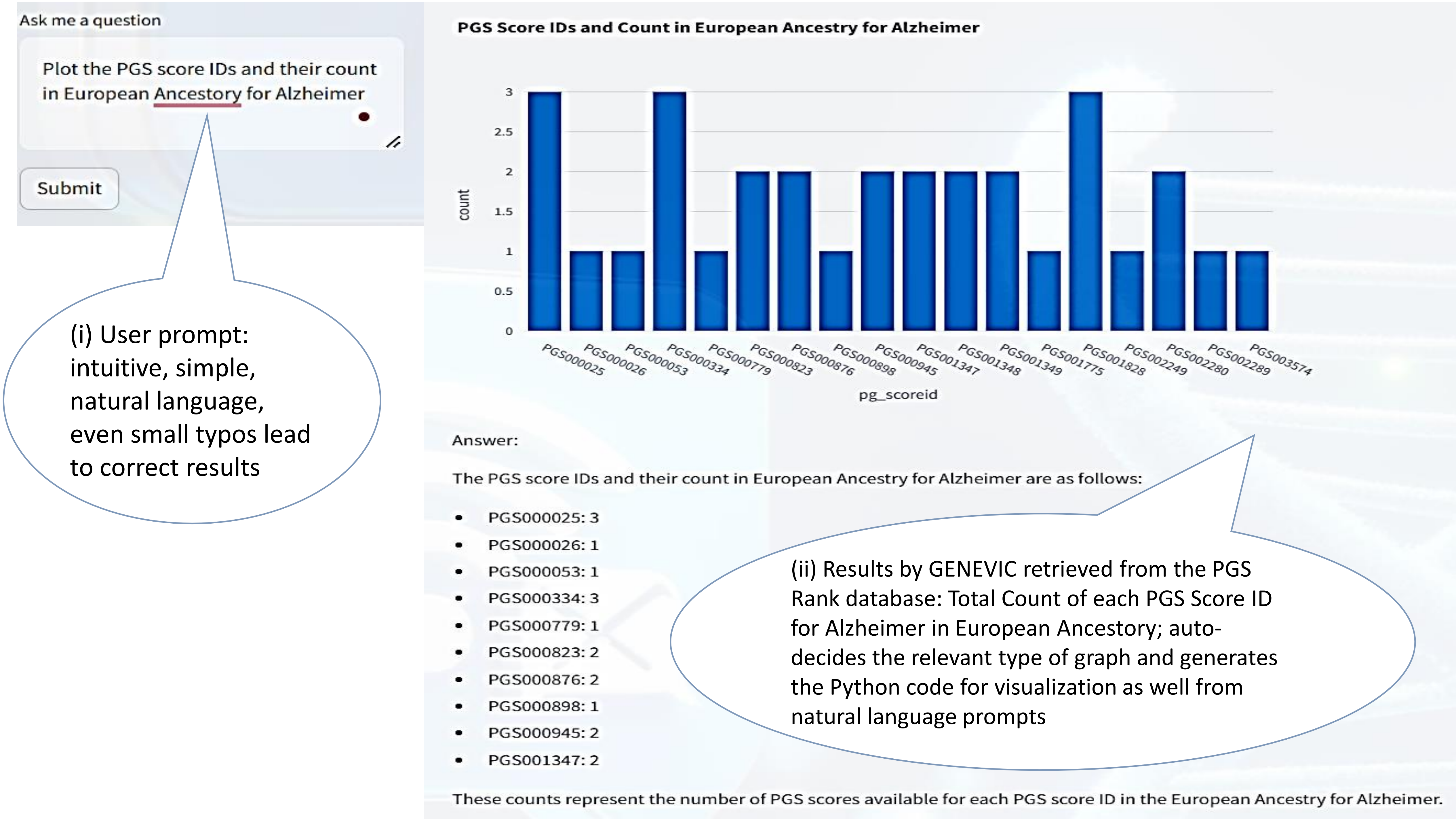
Ask me a question
Plot the PGS score IDs and their count in European Ancestory for Alzheimer
Submit
(i) User prompt: intuitive, simple, natural language, even small typos lead to correct results
PGS Score IDs and Count in European Ancestry for Alzheimer
count
0
0.5
1
1.5
2
2.5
3
PGS000025
PGS000026
PGS000053
PGS000334
PGS000779
PGS000823
PGS000876
PGS000898
PGS000945
PGS001347
PGS001348
PGS001349
PGS001775
PGS001828
PGS002249
PGS002280
PGS002289
PGS003574
pg_scoreid
Answer:
The PGS score IDs and their count in European Ancestry for Alzheimer are as follows:
PGS000025: 3
PGS000026: 1
PGS000053: 1
PGS000334: 3
PGS000779: 1
PGS000823: 2
PGS000876: 2
PGS000898: 1
PGS000945: 2
PGS001347: 2
These counts represent the number of PGS scores available for each PGS score ID in the European Ancestry for Alzheimer.
(ii) Results by GENEVIC retrieved from the PGS Rank database: Total Count of each PGS Score ID for Alzheimer in European Ancestory; auto-decides the relevant type of graph and generates the Python code for visualization as well from natural language prompts

PGS Chat
Test Case 5: Query ChatGPT Directly

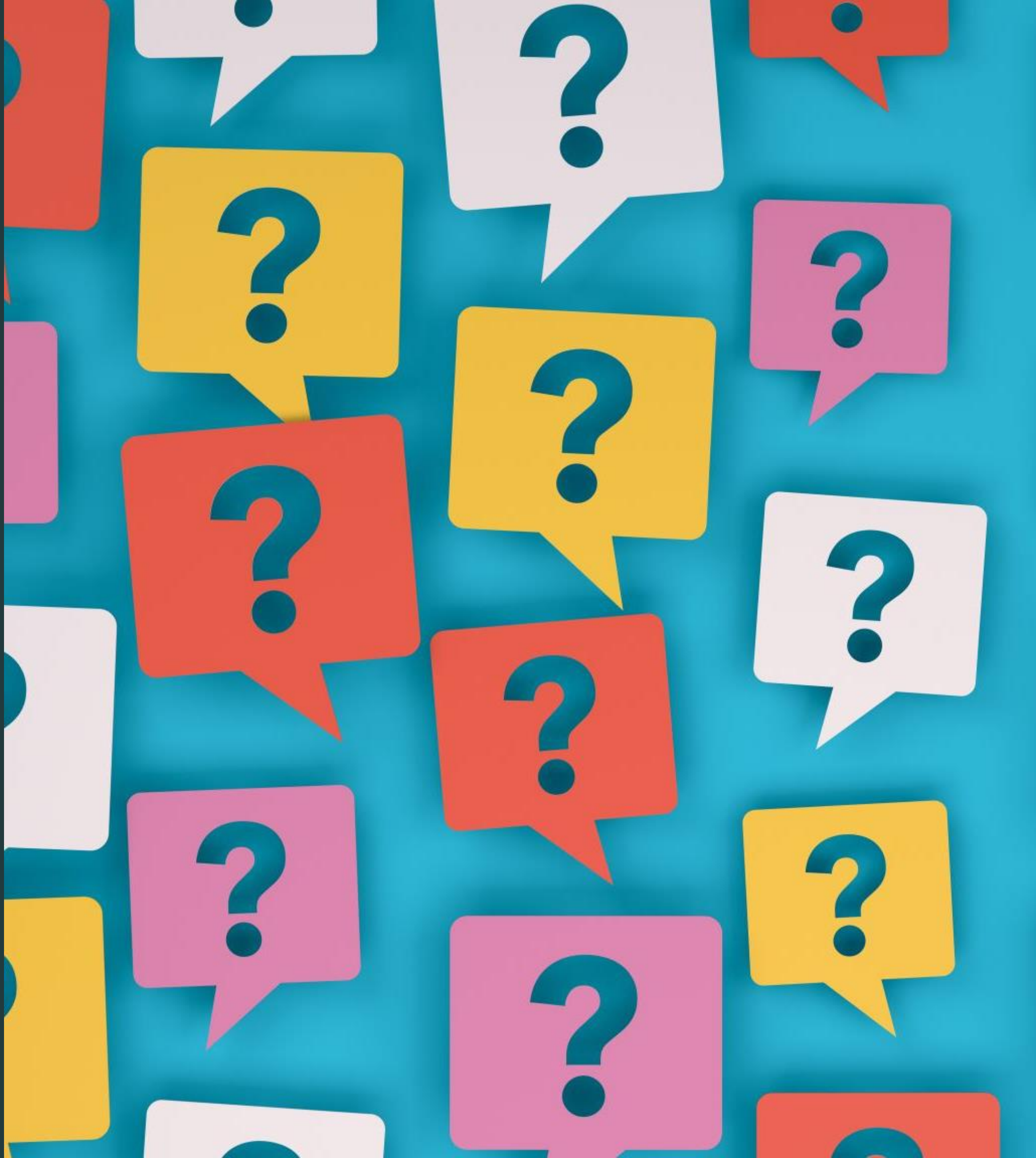

Query ChatGPT directly

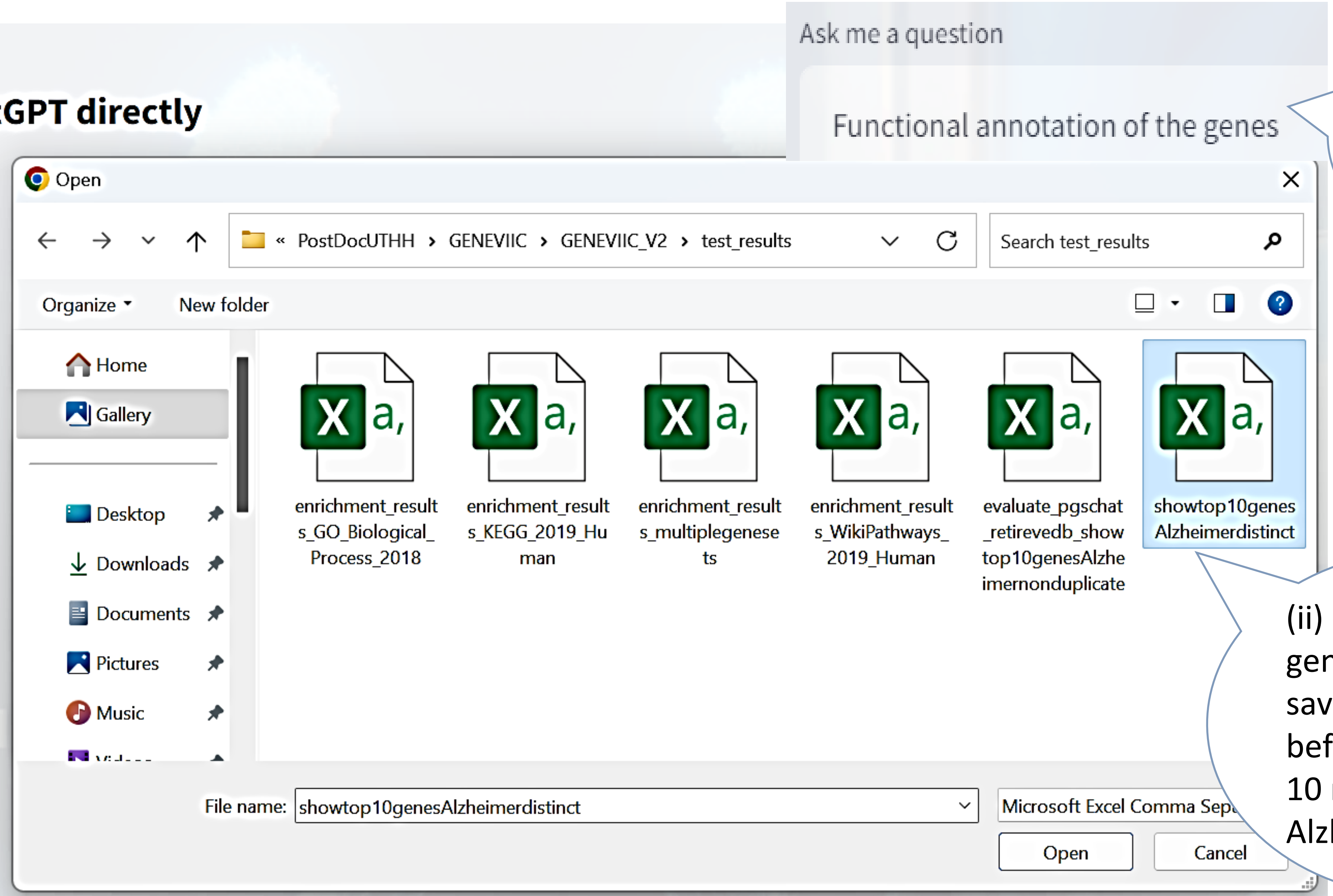

(i) User prompt: *Functional annotation of the genes*

(ii) Upload any gene list (e.g. saved csv file from before showing top 10 ranked genes of Alzheimer's)

..Contd..

Functional annotation is the process of assigning biological functions to genes based on experimental evidence or computational predictions. Here is a brief functional annotation of the genes you mentioned:

1. TREM2 (Triggering Receptor Expressed on Myeloid Cells 2): It is involved in the regulation of immune responses and phagocytosis. Mutations in TREM2 have been associated with increased risk of Alzheimer's disease.

2. SORL1 (Sortilin-Related Receptor 1): It plays a role in the trafficking and processing of amyloid precursor protein (APP), which is involved in the production of amyloid-beta plaques in Alzheimer's disease.

3. APOC1 (Apolipoprotein C1): It is a component of lipoprotein particles and plays a role in lipid metabolism. Variations in APOC1 have been associated with increased risk of Alzheimer's disease.

4. APOE (Apolipoprotein E): It is involved in lipid transport and metabolism. APOE has been extensively studied in relation to Alzheimer's disease, as certain variants of this gene are associated with increased risk.

5. BIN1 (Bridging Integrator 1): It is involved in membrane remodeling and endocytosis. BIN1 has been implicated in Alzheimer's disease, as it interacts with tau protein and affects its aggregation.

6. CYP27C1 (Cytochrome P450 Family 27 Subfamily C Member 1): It encodes an enzyme involved in the metabolism of vitamin D. Its specific role in relation to Alzheimer's disease is not well understood.

7. ABI3 (Abl Interactor 3): It is involved in signal transduction and regulation of cell adhesion. ABI3 has been associated with immune-related disorders, but its role in Alzheimer's disease is not well characterized.

8. DSG2 (Desmoglein 2): It is a component of desmosomes, which are cell adhesion structures. DSG2 mutations have been associated with arrhythmogenic right ventricular cardiomyopathy, but its role in Alzheimer's disease is unclear.

Please note that the functional annotations provided here are a general overview and may not encompass all known functions or associations of these genes. Further research and experimental evidence are required to fully understand the functions and roles of these genes in specific biological processes or diseases.

(iii) Chat GPT/GPT-4 provides the answer to the query using its vast pre-trained knowledge

Gene API Chat

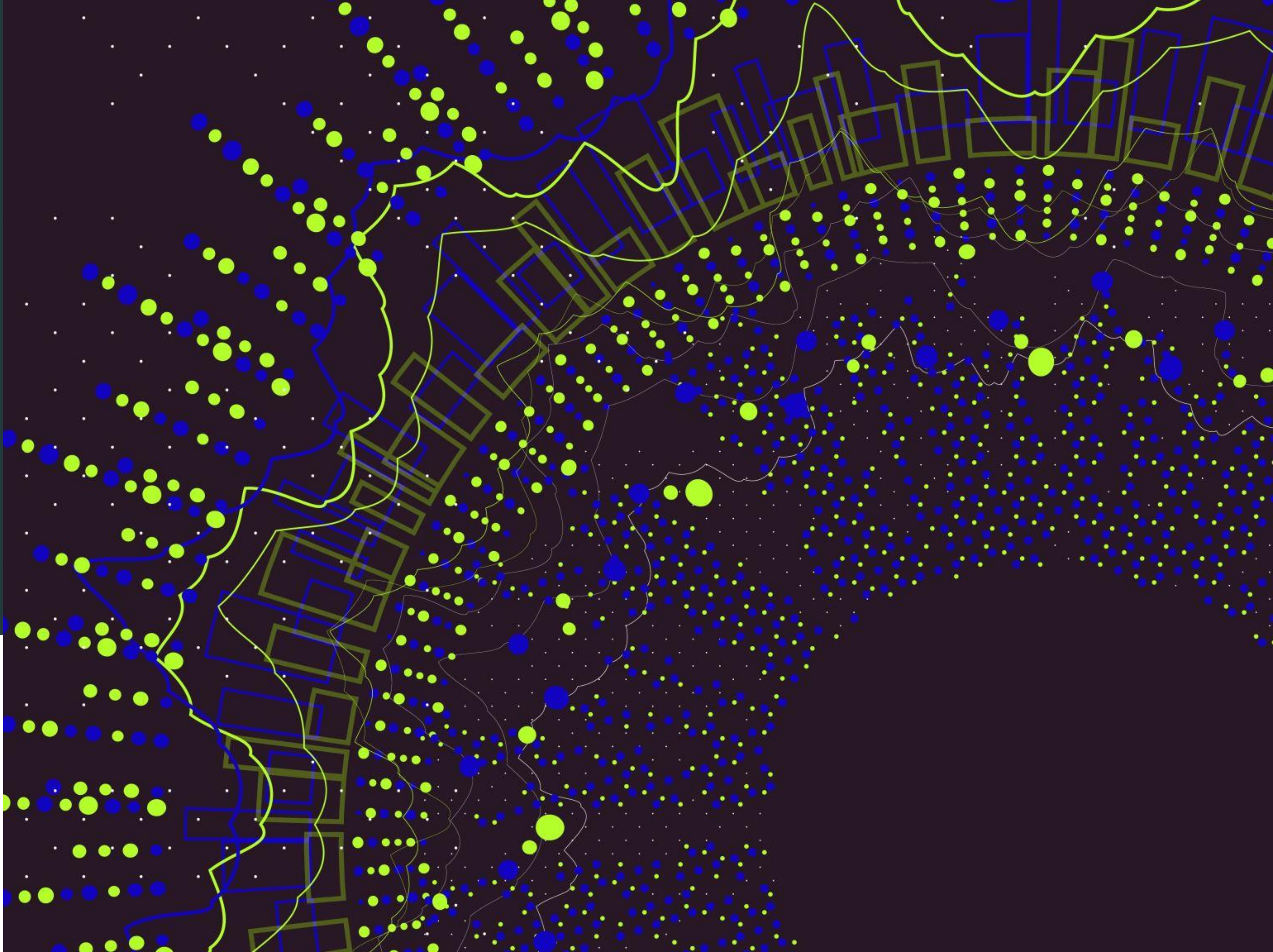

GeneAPI Chat Test Case 1: STRING API

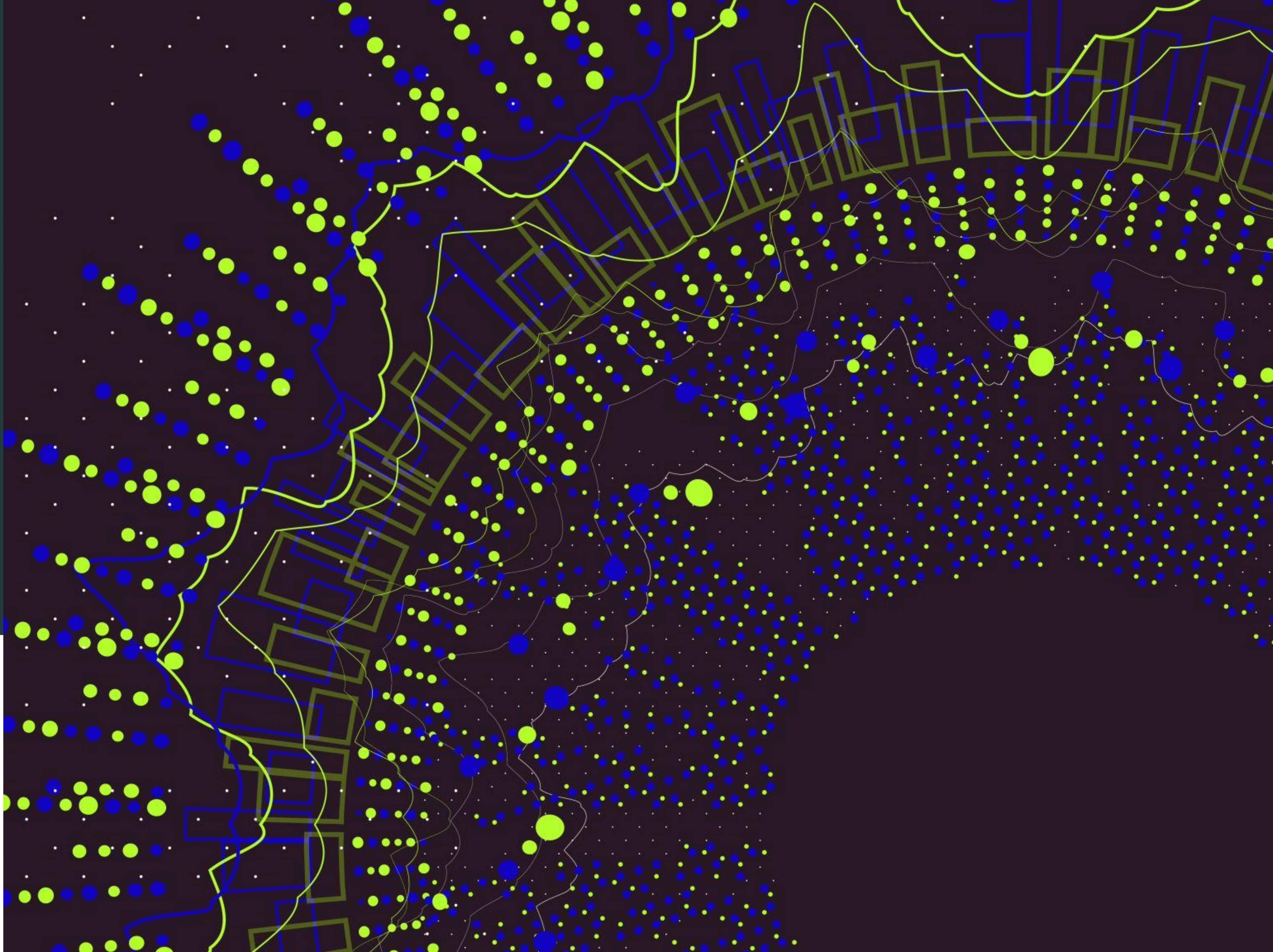

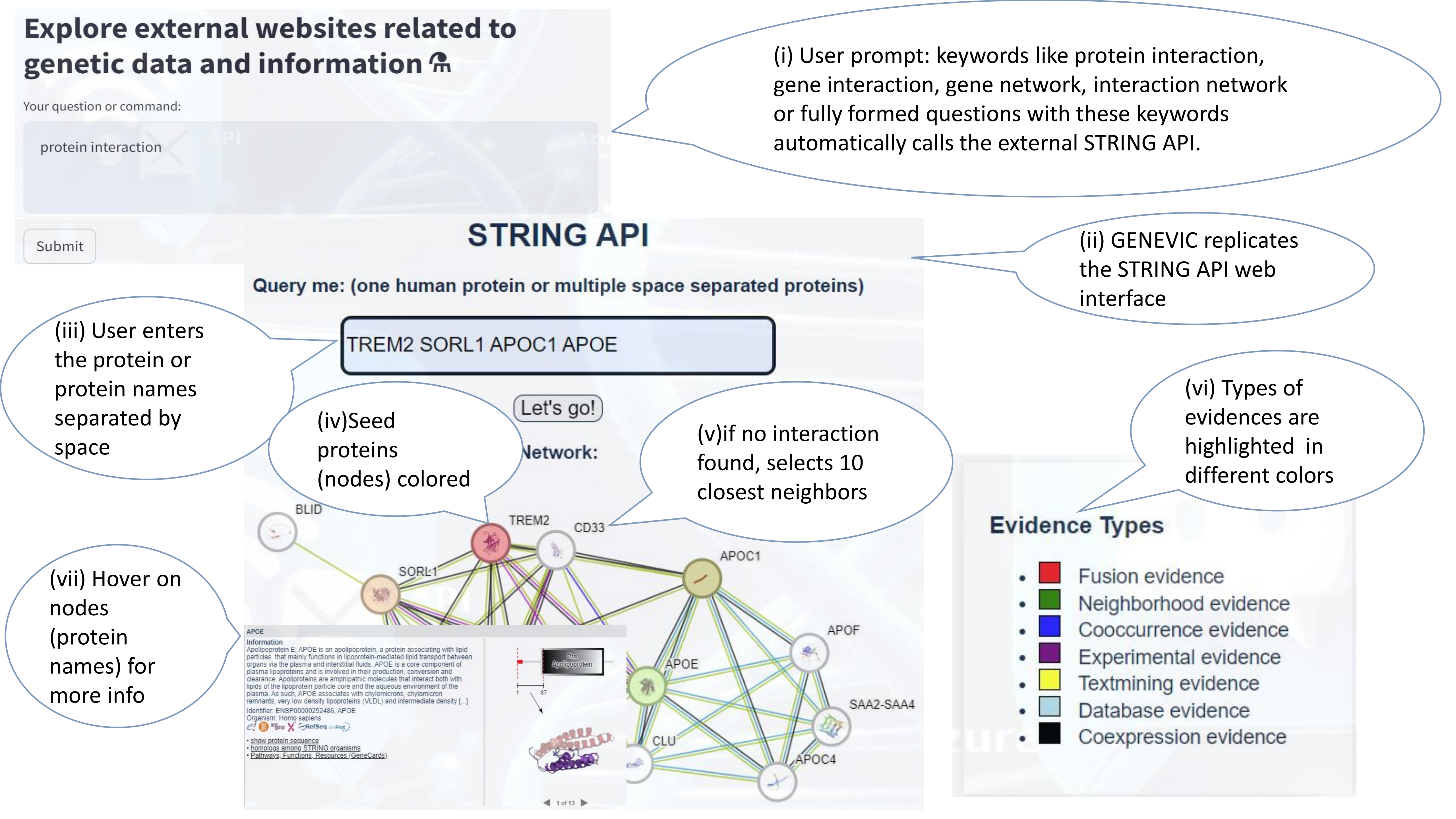

Explore external websites related to genetic data and information
Your question or command:
protein interaction
Submit
(i) User prompt: keywords like protein interaction, gene interaction, gene network, interaction network or fully formed questions with these keywords automatically calls the external STRING API.
STRING API
(ii) GENEVIC replicates the STRING API web interface
Query me: (one human protein or multiple space separated proteins)
TREM2 SORL1 APOC1 APOE
(iii) User enters the protein or protein names separated by space
Let's go!
(iv)Seed proteins (nodes) colored
Network:
(v)if no interaction found, selects 10 closest neighbors
(vi) Types of evidences are highlighted in different colors
BLID
TREM2
CD33
SORL1
APOC1
APOF
APOE
SAA2-SAA4
CLU
APOC4
(vii) Hover on nodes (protein names) for more info
APOE
Information
Apolipoprotein E; APOE is an apolipoprotein, a protein associating with lipid particles, that mainly functions in lipoprotein-mediated lipid transport between organs via the plasma and interstitial fluids. APOE is a core component of plasma lipoproteins and is involved in their production, conversion and clearance. Apoliproteins are amphipathic molecules that interact both with lipids of the lipoprotein particle core and the aqueous environment of the plasma. As such, APOE associates with chylomicrons, chylomicron remnants, very low density lipoproteins (VLDL) and intermediate density [...]
Identifier: ENSP00000252486, APOE
Organism: Homo sapiens
RefSeq
• show protein sequence
• homologs among STRING organisms
• Pathways, Functions, Resources (GeneCards)
Apolipoprotein
1 of 13
Evidence Types
Fusion evidence
Neighborhood evidence
Cooccurrence evidence
Experimental evidence
Textmining evidence
Database evidence
Coexpression evidence

GeneAPI Chat Test Case 2: ENRICHR API

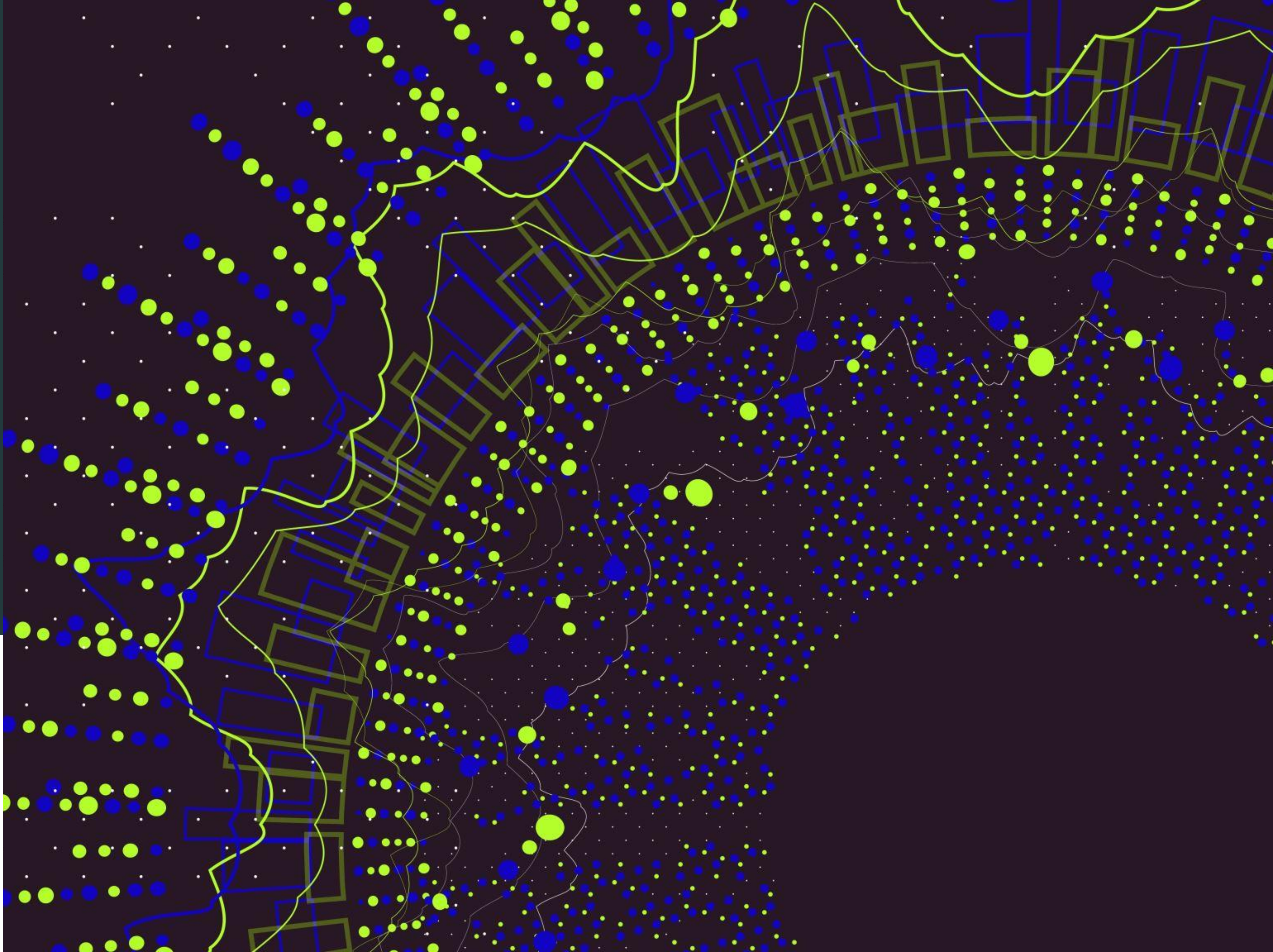

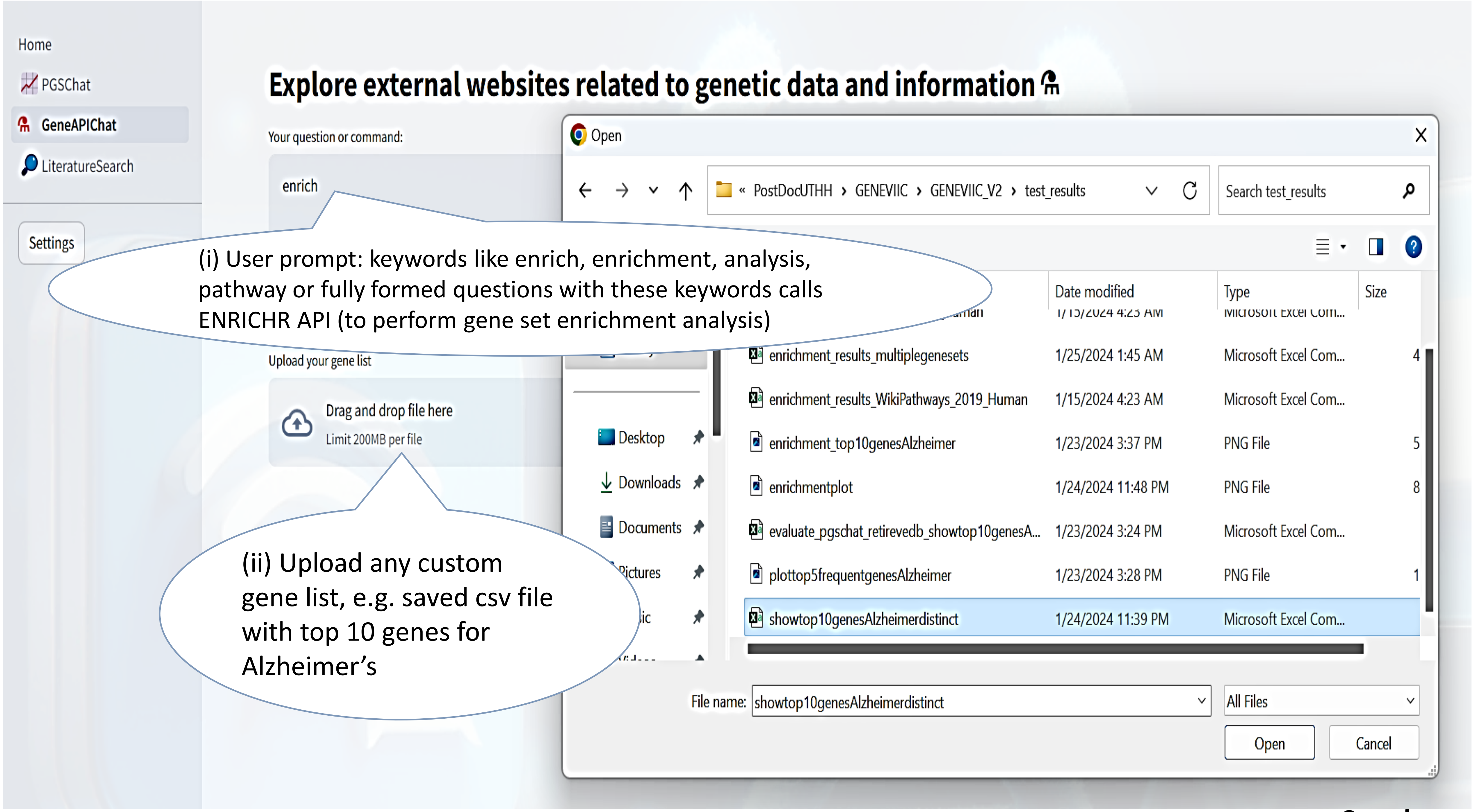
Home
PGSChat
GeneAPIChat
LiteratureSearch
Settings
Explore external websites related to genetic data and information
Your question or command:
enrich
(i) User prompt: keywords like enrich, enrichment, analysis, pathway or fully formed questions with these keywords calls ENRICHR API (to perform gene set enrichment analysis)
Upload your gene list
Drag and drop file here
Limit 200MB per file
(ii) Upload any custom gene list, e.g. saved csv file with top 10 genes for Alzheimer's
Open
« PostDocUTHH › GENEVIIC › GENEVIIC_V2 › test_results
Search test_results
Date modified
Type
Size
enrichment_results_multiplegenesets 1/25/2024 1:45 AM Microsoft Excel Com...
enrichment_results_WikiPathways_2019_Human 1/15/2024 4:23 AM Microsoft Excel Com...
enrichment_top10genesAlzheimer 1/23/2024 3:37 PM PNG File
enrichmentplot 1/24/2024 11:48 PM PNG File
evaluate_pgschat_retirevedb_showtop10genesA... 1/23/2024 3:24 PM Microsoft Excel Com...
plottop5frequentgenesAlzheimer 1/23/2024 3:28 PM PNG File
showtop10genesAlzheimerdistinct 1/24/2024 11:39 PM Microsoft Excel Com...
Desktop
Downloads
Documents
File name: showtop10genesAlzheimerdistinct
All Files
Open
Cancel

..Contd..

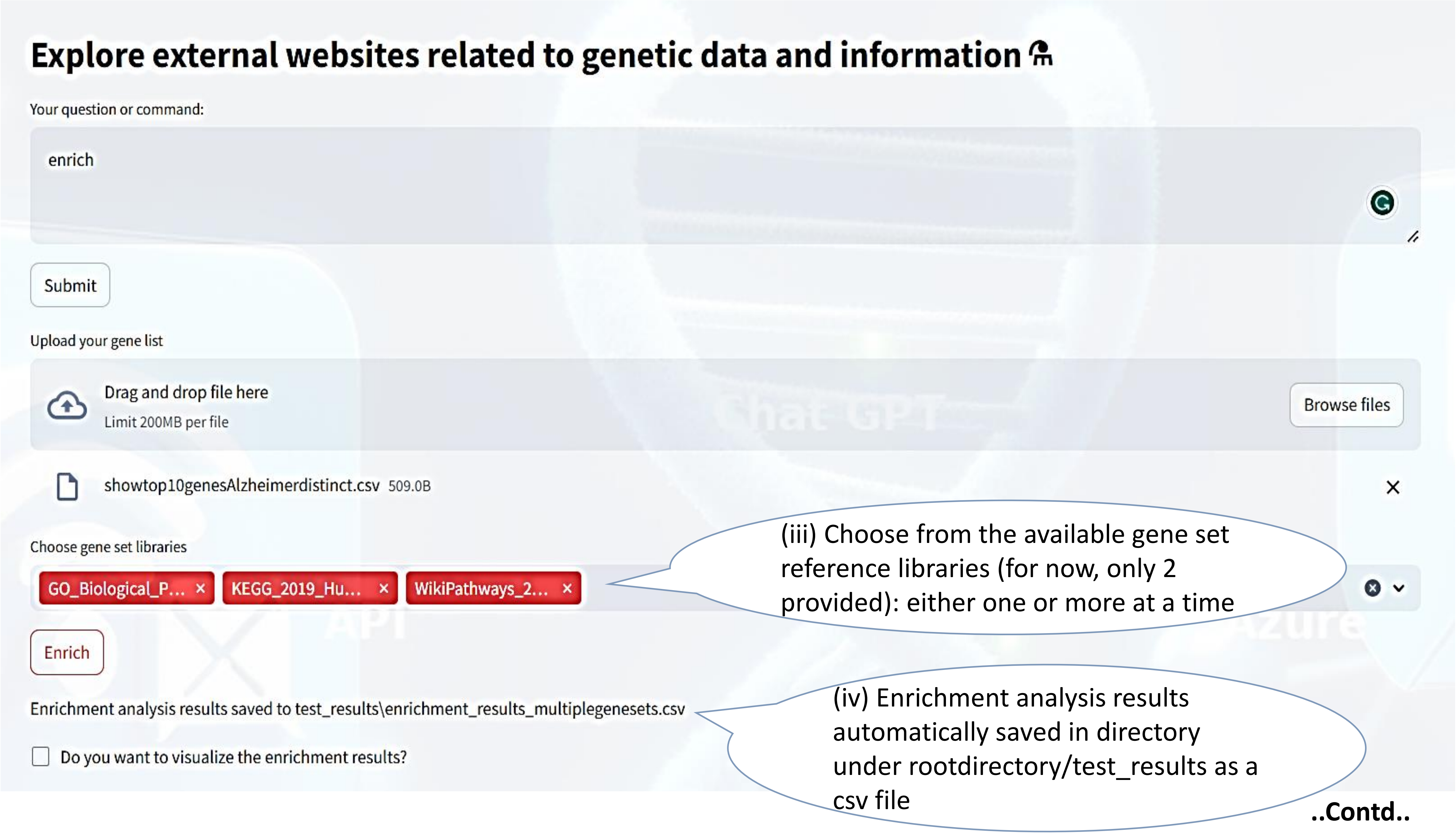
Explore external websites related to genetic data and information
Your question or command:
enrich
Submit
Upload your gene list
Drag and drop file here
Limit 200MB per file
Browse files
showtop10genesAlzheimerdistinct.csv 509.0B
Choose gene set libraries
GO_Biological_P...
KEGG_2019_Hu...
WikiPathways_2...
Enrich
Enrichment analysis results saved to test_results\enrichment_results_multiplegenesets.csv
Do you want to visualize the enrichment results?
(iii) Choose from the available gene set reference libraries (for now, only 2 provided): either one or more at a time
(iv) Enrichment analysis results automatically saved in directory under rootdirectory/test_results as a csv file

..Contd..

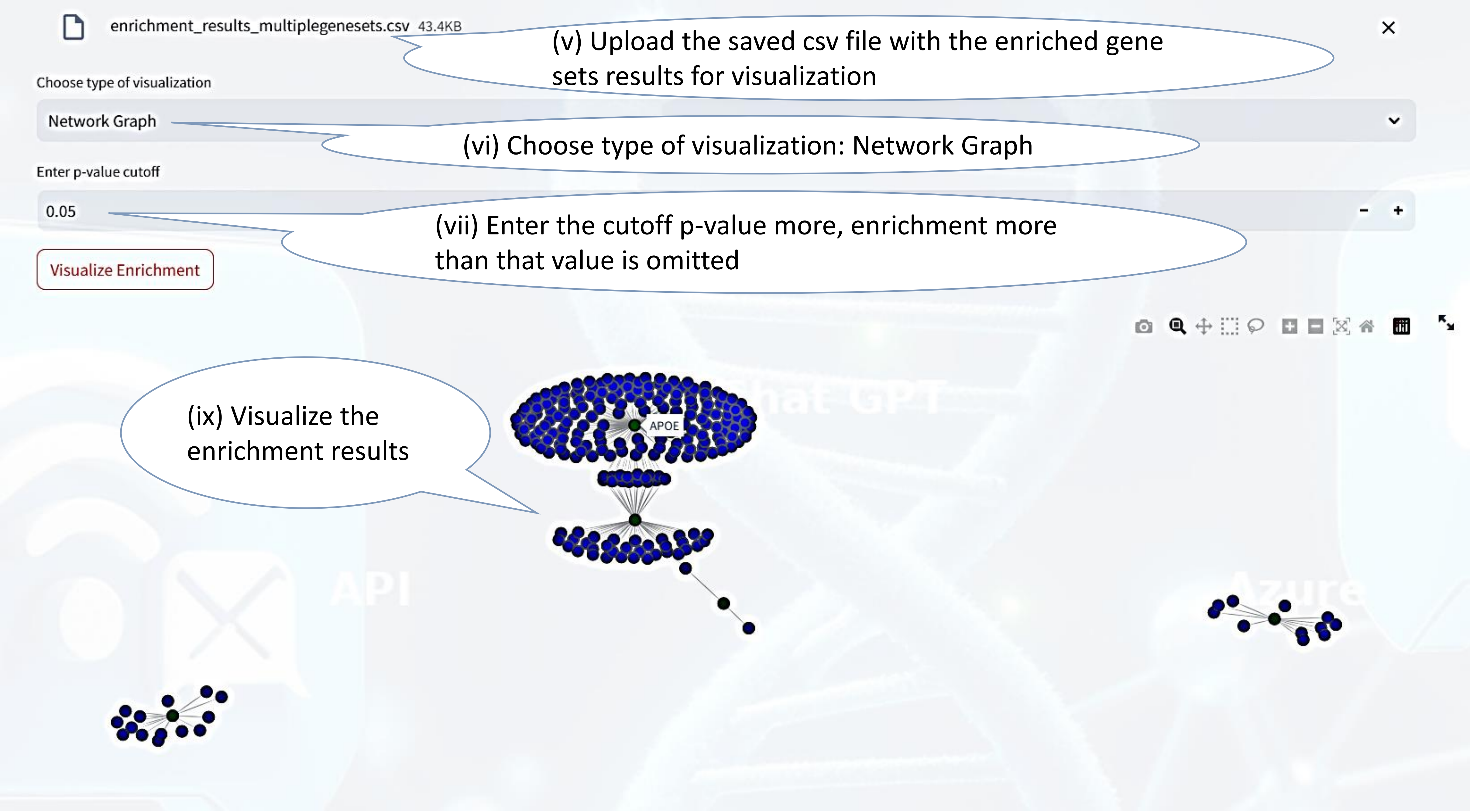
enrichment_results_multiplegenesets.csv 43.4KB
(v) Upload the saved csv file with the enriched gene sets results for visualization
Choose type of visualization
Network Graph
(vi) Choose type of visualization: Network Graph
Enter p-value cutoff
0.05
(vii) Enter the cutoff p-value more, enrichment more than that value is omitted
Visualize Enrichment
(ix) Visualize the enrichment results
APOE

Literature Search

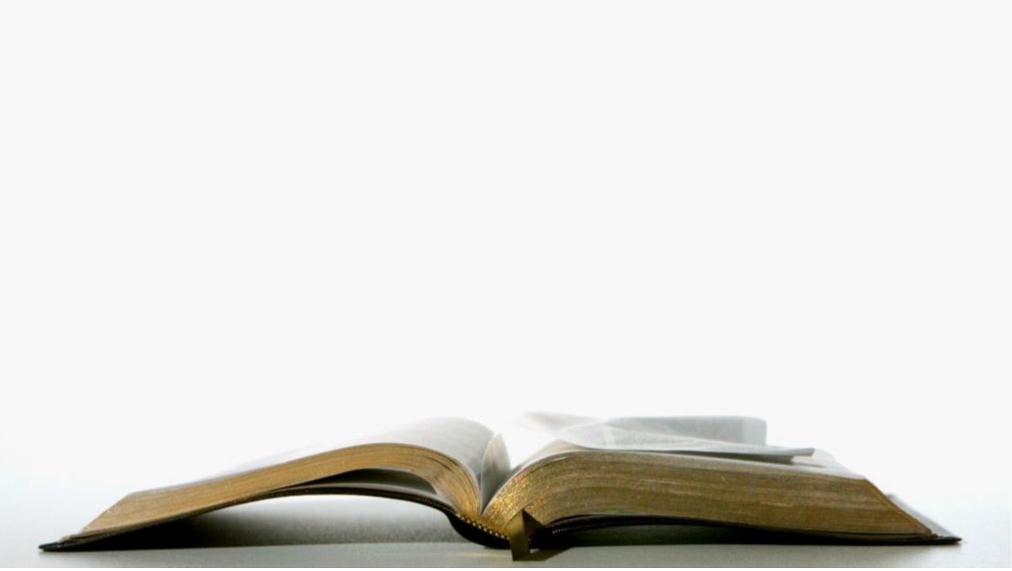

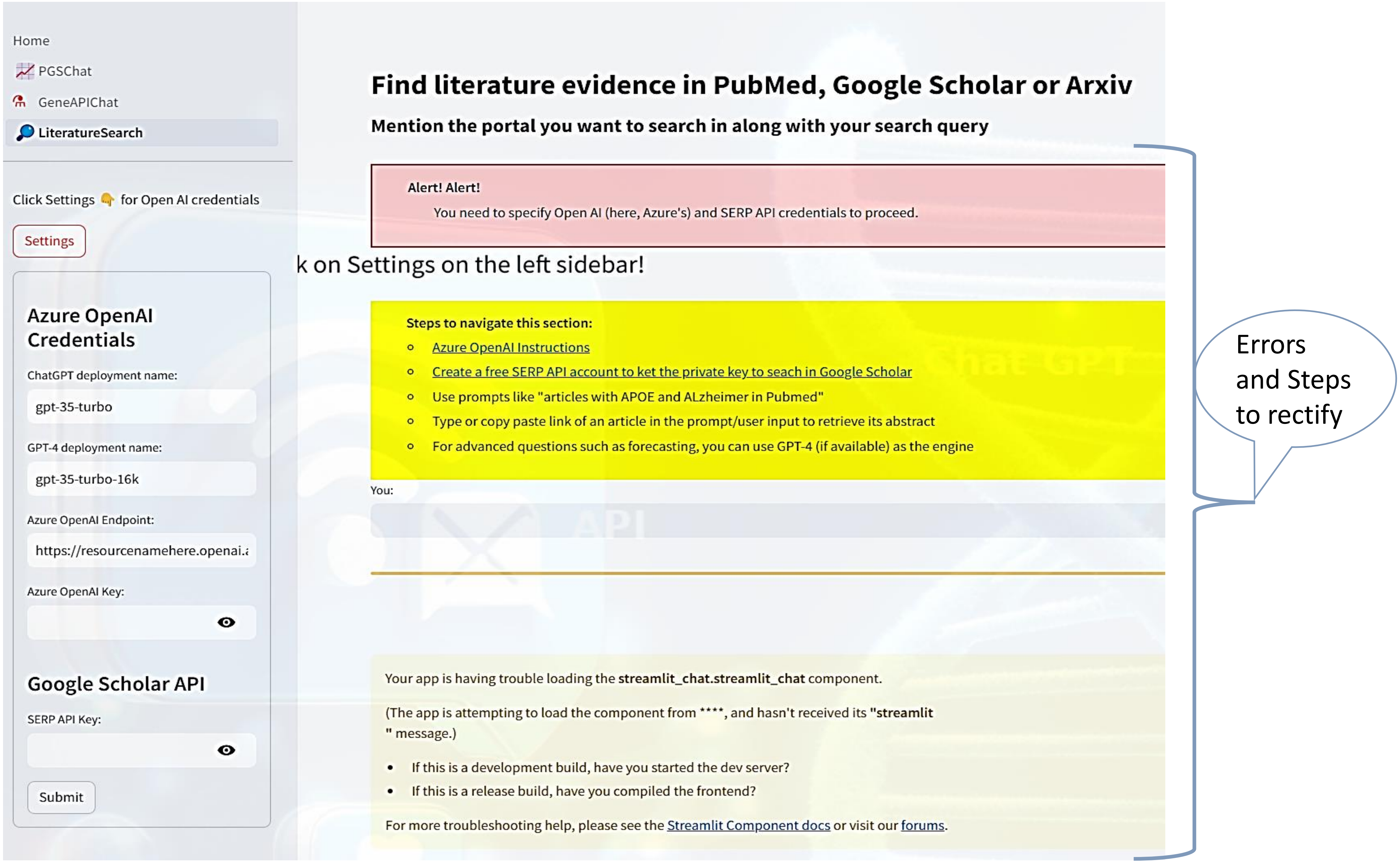
Home
PGSChat
GeneAPIChat
LiteratureSearch
Click Settings for Open AI credentials
Settings
Azure OpenAI Credentials
ChatGPT deployment name:
gpt-35-turbo
GPT-4 deployment name:
gpt-35-turbo-16k
Azure OpenAI Endpoint:
https://resourcenamehere.openai.
Azure OpenAI Key:
Google Scholar API
SERP API Key:
Submit
Find literature evidence in PubMed, Google Scholar or Arxiv
Mention the portal you want to search in along with your search query
Alert! Alert!
You need to specify Open AI (here, Azure's) and SERP API credentials to proceed.
k on Settings on the left sidebar!
Steps to navigate this section:
Azure OpenAI Instructions
Create a free SERP API account to ket the private key to seach in Google Scholar
Use prompts like "articles with APOE and ALzheimer in Pubmed"
Type or copy paste link of an article in the prompt/user input to retrieve its abstract
For advanced questions such as forecasting, you can use GPT-4 (if available) as the engine
You:
Your app is having trouble loading the streamlit_chat.streamlit_chat component.
(The app is attempting to load the component from ****, and hasn't received its "streamlit
" message.)
If this is a development build, have you started the dev server?
If this is a release build, have you compiled the frontend?
For more troubleshooting help, please see the Streamlit Component docs or visit our forums.
Errors and Steps to rectify

Literature Search
Test Case 1: Search in PubMed

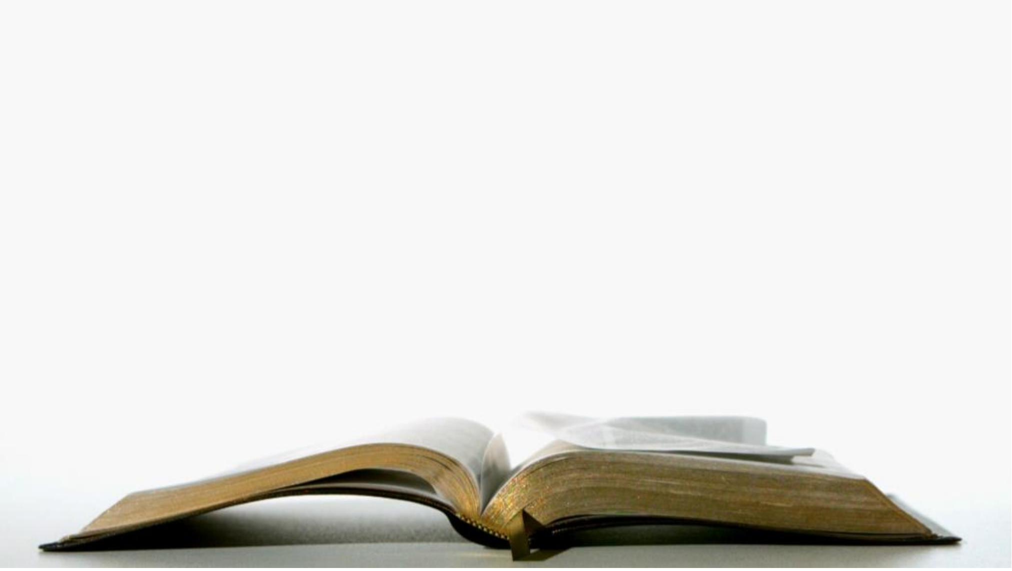

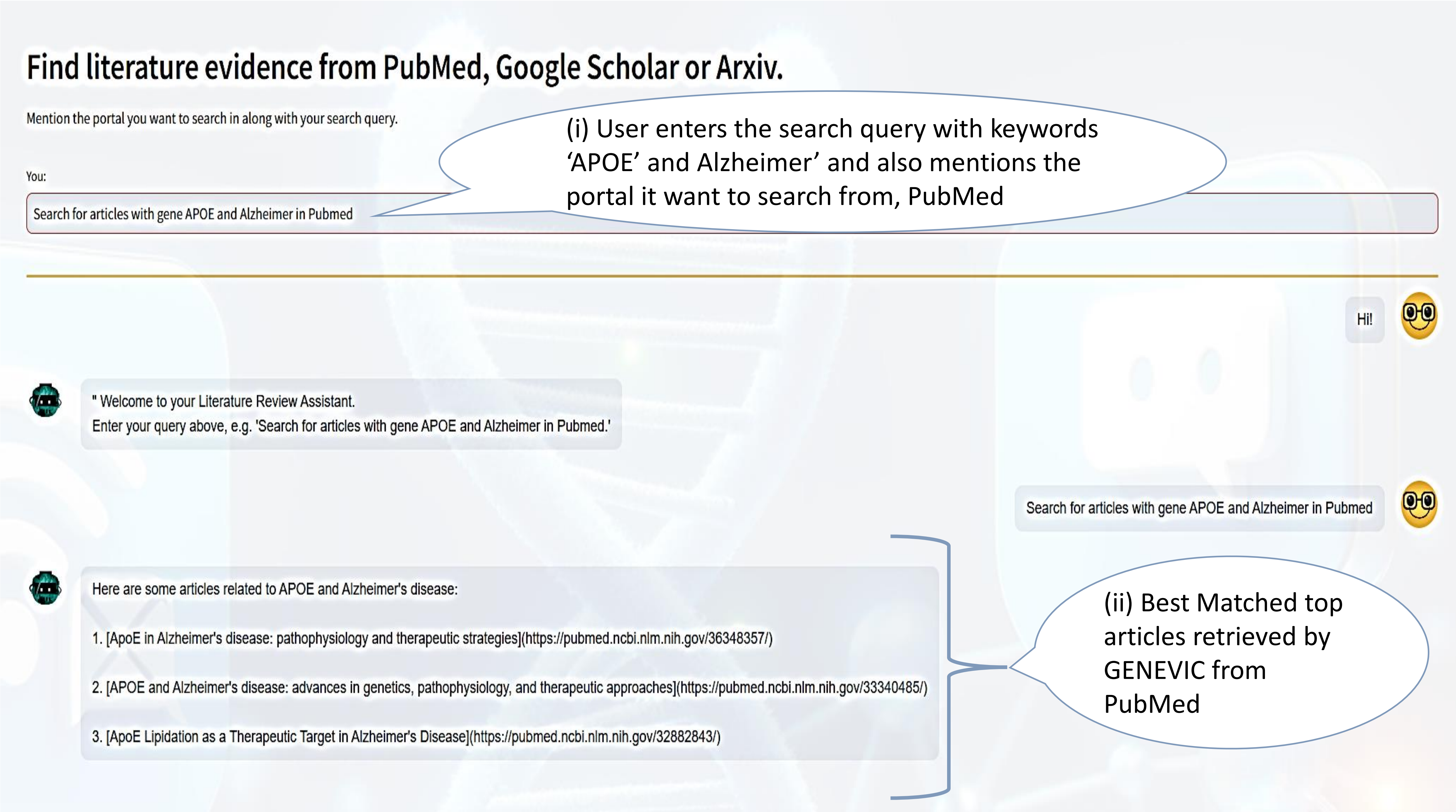
Find literature evidence from PubMed, Google Scholar or Arxiv.
Mention the portal you want to search in along with your search query.
You:
Search for articles with gene APOE and Alzheimer in Pubmed
(i) User enters the search query with keywords ‘APOE’ and Alzheimer’ and also mentions the portal it want to search from, PubMed
Hi!
" Welcome to your Literature Review Assistant.
Enter your query above, e.g. 'Search for articles with gene APOE and Alzheimer in Pubmed.'
Search for articles with gene APOE and Alzheimer in Pubmed
Here are some articles related to APOE and Alzheimer's disease:
1. [ApoE in Alzheimer's disease: pathophysiology and therapeutic strategies](https://pubmed.ncbi.nlm.nih.gov/36348357/)
2. [APOE and Alzheimer's disease: advances in genetics, pathophysiology, and therapeutic approaches](https://pubmed.ncbi.nlm.nih.gov/33340485/)
3. [ApoE Lipidation as a Therapeutic Target in Alzheimer's Disease](https://pubmed.ncbi.nlm.nih.gov/32882843/)
(ii) Best Matched top articles retrieved by GENEVIC from PubMed

Literature Search
Test Case 2: Search in Google Scholar and ArXiv

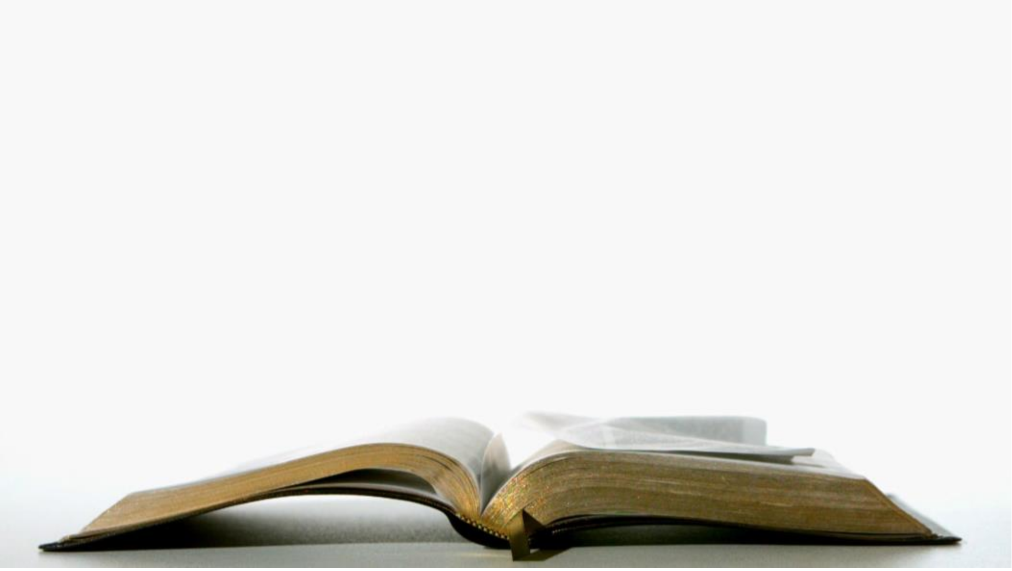

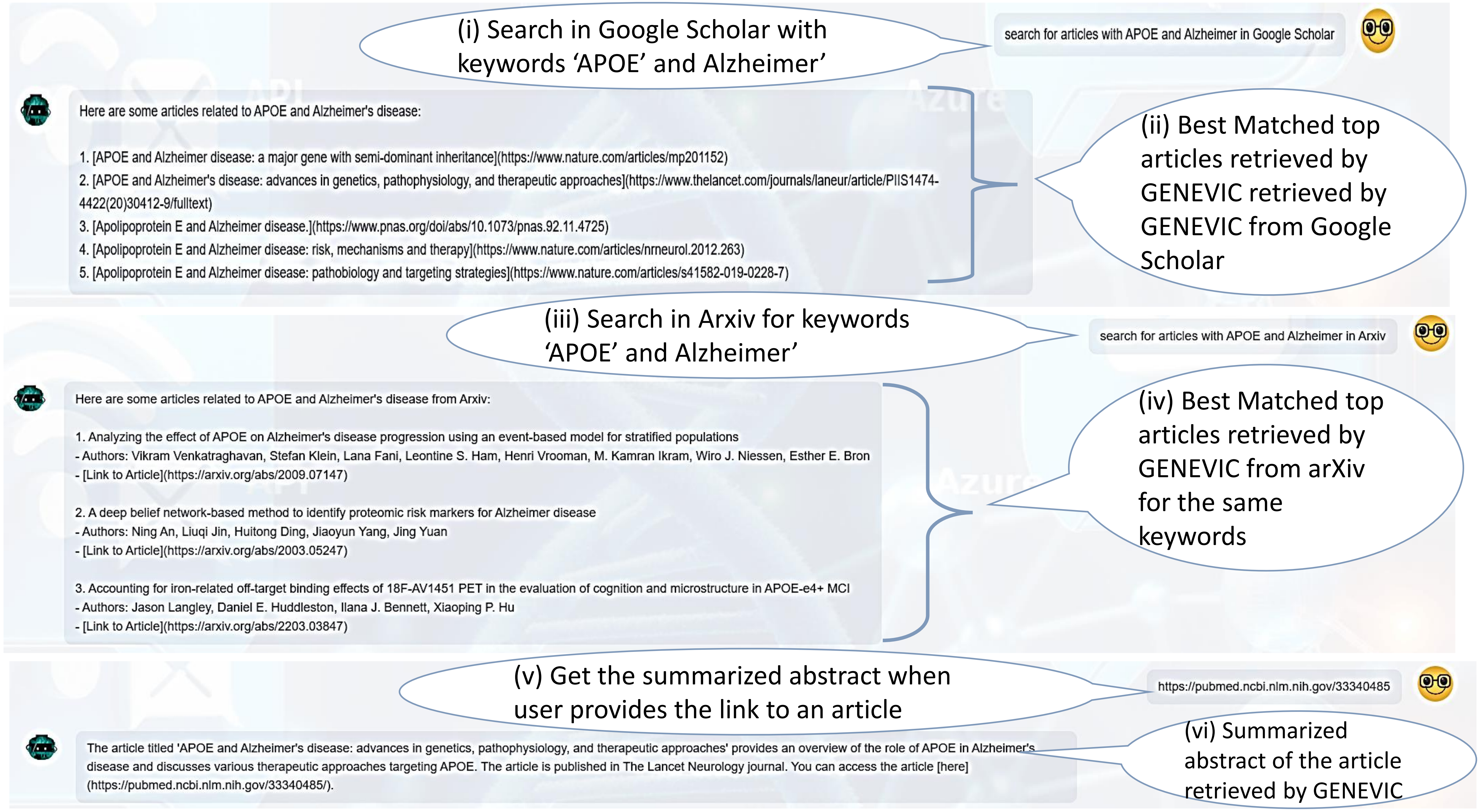
(i) Search in Google Scholar with keywords 'APOE' and Alzheimer'
search for articles with APOE and Alzheimer in Google Scholar
Here are some articles related to APOE and Alzheimer's disease:
1. [APOE and Alzheimer disease: a major gene with semi-dominant inheritance](https://www.nature.com/articles/mp201152)
2. [APOE and Alzheimer's disease: advances in genetics, pathophysiology, and therapeutic approaches](https://www.thelancet.com/journals/laneur/article/PIIS1474-4422(20)30412-9/fulltext)
3. [Apolipoprotein E and Alzheimer disease.](https://www.pnas.org/doi/abs/10.1073/pnas.92.11.4725)
4. [Apolipoprotein E and Alzheimer disease: risk, mechanisms and therapy](https://www.nature.com/articles/nrneurol.2012.263)
5. [Apolipoprotein E and Alzheimer disease: pathobiology and targeting strategies](https://www.nature.com/articles/s41582-019-0228-7)
(ii) Best Matched top articles retrieved by GENEVIC retrieved by GENEVIC from Google Scholar
(iii) Search in Arxiv for keywords 'APOE' and Alzheimer'
search for articles with APOE and Alzheimer in Arxiv
Here are some articles related to APOE and Alzheimer's disease from Arxiv:
1. Analyzing the effect of APOE on Alzheimer's disease progression using an event-based model for stratified populations
- Authors: Vikram Venkatraghavan, Stefan Klein, Lana Fani, Leontine S. Ham, Henri Vrooman, M. Kamran Ikram, Wiro J. Niessen, Esther E. Bron
- [Link to Article](https://arxiv.org/abs/2009.07147)
2. A deep belief network-based method to identify proteomic risk markers for Alzheimer disease
- Authors: Ning An, Liuqi Jin, Huitong Ding, Jiaoyun Yang, Jing Yuan
- [Link to Article](https://arxiv.org/abs/2003.05247)
3. Accounting for iron-related off-target binding effects of 18F-AV1451 PET in the evaluation of cognition and microstructure in APOE-e4+ MCI
- Authors: Jason Langley, Daniel E. Huddleston, Ilana J. Bennett, Xiaoping P. Hu
- [Link to Article](https://arxiv.org/abs/2203.03847)
(iv) Best Matched top articles retrieved by GENEVIC from arXiv for the same keywords
(v) Get the summarized abstract when user provides the link to an article
https://pubmed.ncbi.nlm.nih.gov/33340485
The article titled 'APOE and Alzheimer's disease: advances in genetics, pathophysiology, and therapeutic approaches' provides an overview of the role of APOE in Alzheimer's disease and discusses various therapeutic approaches targeting APOE. The article is published in The Lancet Neurology journal. You can access the article [here](https://pubmed.ncbi.nlm.nih.gov/33340485/).
(vi) Summarized abstract of the article retrieved by GENEVIC

Mitigation of Hallucination

GENEVIC leverages a **Domain-Specific Retrieval Augmented Generation (RAG**) to enhance factual accuracy and mitigate hallucinations. This involves integrating LLMs with **domain-specific databases** and **external sources**, enabling the tool to generate responses grounded in real-time, verified information. This method ensures, as is described below, that novel findings are backed by external, authoritative data sources to ensure the accuracy and reliability of the information it generates:

1. **Curated Database**: GENEVIC's capability to analyze any user-uploaded database allows for a robust cross-referencing mechanism. By querying well-validated and curated datasets, the tool can provide results grounded in factual data, reducing the risk of LLM hallucinations.
2. **External API Integration**: The integration of STRING and ENRICHR APIs automates the retrieval of highly reliable bioinformatics data. STRING provides known and predicted protein-protein interactions, while ENRICHR offers comprehensive gene set enrichment analysis. These APIs are based on extensive, peer-reviewed research, ensuring that the data used for analysis is credible.
3. **Literature Search**: GENEVIC supports literature searches from reputable sources such as PubMed, Google Scholar, and arXiv. This feature allows users to validate findings by cross-referencing them with published scientific literature. The tool automatically pulls in relevant paper links, summarized or one-line abstracts, and authorship information, enabling users to verify the factual accuracy of the generated information.

Validation of Results

- **Manual Validation**: Cross-referencing GENEVIC's database querying output (via PGSChat interface) with those generated when the same SQL code is run manually in a popular database IDE, e.g., SQL Studio.
- **Source Verification**: Ensuring that the data retrieved from APIs like STRING and ENRICHR, as well as from online literature sites, are accurate by validating these outputs of GENEVIC against their original sources for the same input prompt/search query.
- **Percentage of Factual Correctness:** By performing one-to-one comparisons, we can calculate the percentage of outputs that match the expected results.

Following this approach, we conducted a preliminary evaluation using 5 test cases each for PGS Chat's Information Retrieval to obtain 90% factual correctness , GeneAPI Chat's STRING API with 100% factual correctness and Literature Search (PubMed, Google Scholar and arXiv) with 95% factual correctness.

For future versions, we plan to conduct more extensive evaluations and incorporate external evaluations to further enhance the reliability and accuracy of the tool. This will involve:

- automated validation techniques
- **Hughes Hallucination Evaluation Model (HHEM)**: a specific metric to detect and quantify hallucinations in the LLM outputs. The Hughes Hallucination Evaluation Model (HHEM) can be adapted to classify the factual consistency of responses, providing a quantifiable measure of reliability. https://huggingface.co/vectara/hallucination_evaluation_model
- feedback from a broader and external user base to continuously improve the system

Pros & Cons

Limitations

- real-time lags
- only a prototype/pilot framework
- demonstrates capabilities using a limited PGS Rank DB
  - ✓data for only three phenotypes
  - ✓rudimentary approach to ranking variants via PGS scores

Contributions

- generative AI unifies and streamlines access, navigation, automate analysis of biomedical DBs and external web APIs
- aids in extracting pivotal genetic variants related to diseases, building an extensive map of variant-gene-trait associations
- intuitive, user-friendly interface
- no biomedical knowledge or technical training required
- leverages standardized databases
- minimal "hallucination"
- customize data sources
- protect sensitive clinical data, Azure OpenAI's HIPAA-compliant infrastructure

Future Directions

- ✓ Better variant ranking scheme:
    - comprehensive data
    - robust weighing scheme
    - various factors:
        - ethnic background
        - genotype data
        - specific PGS scoring techniques
        - degree of sample overlap across PGS datasets for each phenotype
- ✓ broaden the database scope
- ✓ integrate additional biomedical web APIs
- ✓ enhance Literature Search functionalities:

  auto-extraction of deeper insights using advanced NLP techniques
- ✓ integrate automated predictive modeling and analytics using generative AI
