# Supplementary material for: GENEVIC: GENetic data Exploration and Visualization via Intelligent interactive Console
Source: Bioinformatics. 2024 Aug 8;40(10):btae500. doi: 10.1093/bioinformatics/btae500 (PMC11467054; doi:10.1093/bioinformatics/btae500)
Supplement: btae500_Supplementary_Data [file btae500_supplementary_data.zip › Supplementary_LocalInstalaltionGuide.pdf]

Local Installation

Pre-requisites

- Python 3.10+ - **Important**: Python and the pip package manager must be in the path in Windows for the setup scripts to work. - **Important**: Ensure you can run `python --version` from console.\On Ubuntu, you might need to run `sudo apt install python-is-python3` to link `python` to `python3`.
-

Step-wise Instructions

Step 1. Clone this repository

Clone this repository:git clone https://github.com/anath2110/GENEVIC.git
From the terminal, navigate to `cd [path-to-project-root-folder]`

Step 2. Set up enviromental variables

Provide settings for Open AI and Database. You can either create a file named `secrets.env` file in the root of this project folder in your PC as below or do it using the app's GUI later on.

```
- Option 1: use built-in SQLITE. Then you don't need to install SQL Server.

    AZURE_OPENAI_API_KEY="9999999999999999999999999"
    AZURE_OPENAI_GPT4_DEPLOYMENT="NAME_OF_GPT_4_DEPLOYMENT"
    AZURE_OPENAI_CHATGPT_DEPLOYMENT="NAME_OF_CHATGPT_4_DEPLOYMENT"
    AZURE_OPENAI_ENDPOINT=https://openairesourcename.openai.azure.com/
    SQL_ENGINE = "sqlite"


- Option 2: use your own SQL Server

    AZURE_OPENAI_API_KEY="9999999999999999999999999"
    AZURE_OPENAI_ENDPOINT="https://openairesourcename.openai.azure.com/"
    AZURE_OPENAI_GPT4_DEPLOYMENT="NAME_OF_GPT_4_DEPLOYMENT"
    AZURE_OPENAI_CHATGPT_DEPLOYMENT="NAME_OF_CHATGPT_4_DEPLOYMENT"
    SQL_USER="sqluserid"
    SQL_PASSWORD="sqlpassword"
    SQL_DATABASE="WideWorldImportersDW"
    SQL_SERVER="sqlservername.database.windows.net"
```

IMPORTANT If you are a Mac user, please follow this to install ODBC for PYODBC

Step 3. Configure development environment

NOTE all activities in this step will performed using the command line

Step 3.1 Navigate to the root directory of this project

Navigate to `cd [path-to-project-root-folder]`

Step 3.2 Create a python environment

This step is required **ONLY if did not perform this earlier as part of the pre-requisites**

Step 3.3 Import the requirements.txt

Run the command: `pip install -r requirements.txt`

Step 3.4 Run the application locally

To run the application from the command line: `streamlit run app.py`
You will see the application load in your browser.

Note: For troubleshoot, see here **Note**: For Azure Open AI subscription and set up: see here

Docker Installation

Prerequisites:

Install 'Docker' in local system or create an account in Docker Cloud. Help Resources: https://docs.docker.com/engine/install/

Download Docker Image for GENEVIC:

Click here to download the zipped docker image file

Commands:

Run the following commands from the directory where you loaded the above image (here, exmaple for Windows CMD prompt is shown):
docker load -i genevic-v1.tar
This command loads the Docker image from the tar file into your local Docker repository.
docker run -p 8501:8501 genevic-v1
This command runs the container, mapping port 8501 on your local machine to port 8501 in the container.

Web Usage

Access the web application at: https://genevic-anath2024.streamlit.app/
