# Supplementary material for: GENEVIC: GENetic data Exploration and Visualization via Intelligent interactive Console
Source: Bioinformatics. 2024 Aug 8;40(10):btae500. doi: 10.1093/bioinformatics/btae500 (PMC11467054; doi:10.1093/bioinformatics/btae500)
Supplement: btae500_Supplementary_Data [file btae500_supplementary_data.zip › Supplementary_PGSrankDatabase.pdf]

1. PGS Rank Database Schema Diagram

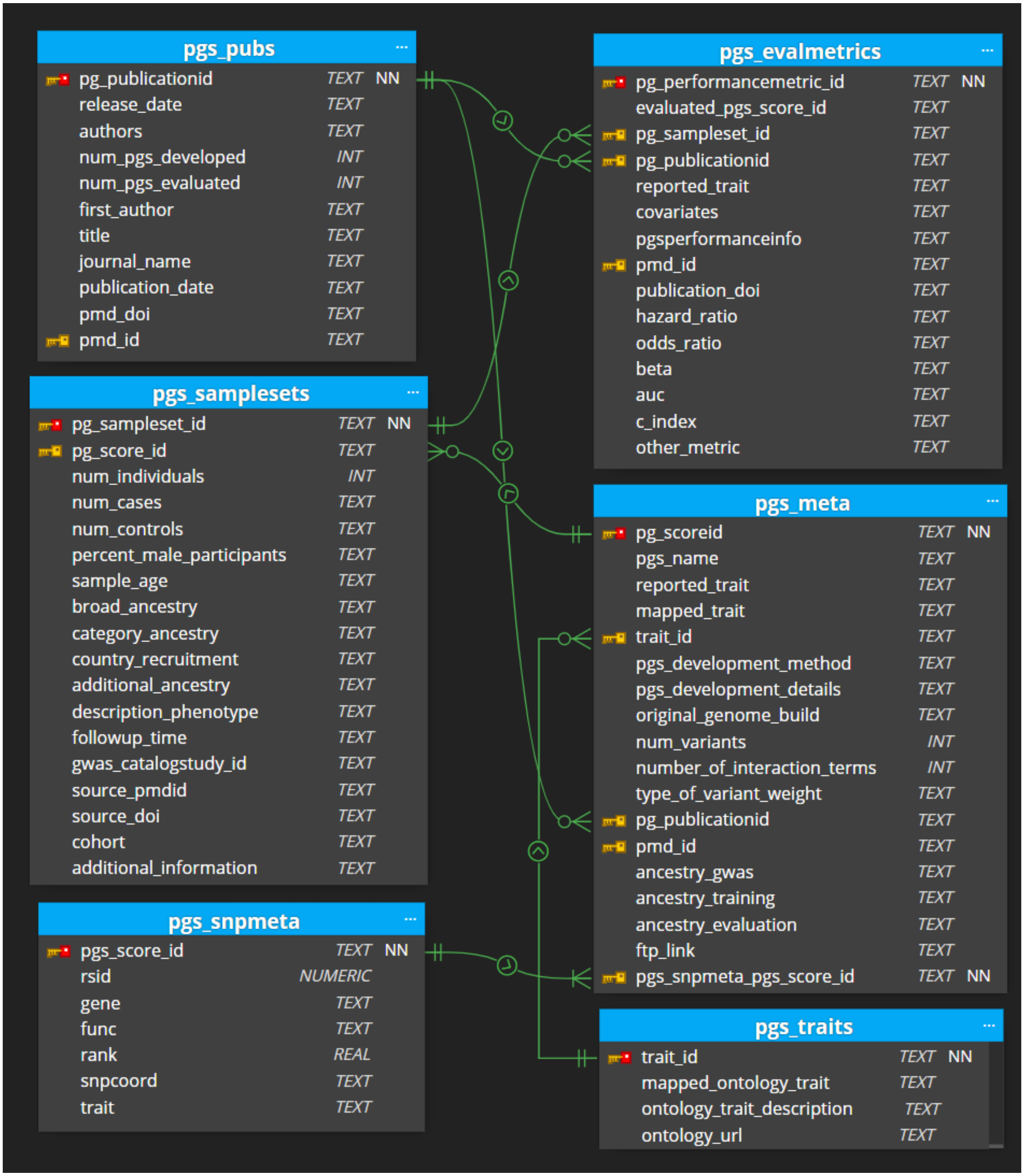

2. Downloadable Links for PGS rank Database

These customized databases, inspired by The Polygenic Score Catalog Website, serve as a pivotal resource and the sample genetic database that is utilized to demo the functionality of the "PGS Chat" interface of the intelligent chat assistant, "GENEVIC". This specialized database is designed to facilitate the exploration and analysis of gene-disease associations, providing researchers with comprehensive variant ranks corresponding to three different phenotypes, , Alzheimer's, schizophrenia, and cognition. The original .csv files used to create the corresponding tables in the database can be downloaded from here. The metadata describing each of the tables and its columns are explained in this website link.

i. Limited version (300 genes, with 100 genes each from 3 traits) :

- Click to download PGS Rank Database(.db format)
- Click to download PGS Rank Database(.zip format)

ii. Original version (all genes from each of the 3 traits):

- Click to download PGS Rank Database(.db format)
- Click to download PGS Rank Database(.zip format)
